# The YTHDC1 reader protein recognizes and regulates the lncRNA MEG3 following its METTL3-mediated m^6^A methylation: a novel mechanism early during radiation-induced liver injury

**DOI:** 10.1038/s41419-025-07417-2

**Published:** 2025-02-24

**Authors:** Gui-yuan Song, Qing-hua Yu, Xue-kun Xing, Xin-ming Fan, Si-guang Xu, Wen-bo Zhang, Yao-yao Wu, Nan Zhang, Tian-zhu Chao, Fei Wang, Cheng-shi Ding, Cun-yang Guo, Li Ma, Chang-ye Sun, Shu-yan Duan, Ping Xu

**Affiliations:** 1 Laboratory of Radiation-induced Diseases and Molecule-targeted Drugs, School of Food and Biomedicine, Zaozhuang University, Zaozhuang, Shandong China; 2 School of Pharmacy, Weifang Medical University, Weifang, Shandong China; 3 School of Public Health, Weifang Medical University, Weifang, Shandong China; 4 School of Public Health, Guilin Medical University, Guilin, Guangxi China; 5 Department of Radiotherapy, Zaozhuang Municipal Hospital, Zaozhuang, Shandong China; 6 Key Laboratory of Medical Tissue Regeneration of Henan Province, Xinxiang Medical University, Xinxiang, Henan China; 7 School of Public Health, Binzhou Medical University, Yantai, Shandong China

**Keywords:** Physiology, Diseases

## Abstract

While apoptotic cell death is known to be central to the pathogenesis of radiation-induced liver injury (RILI), the mechanistic basis for this apoptotic activity remains poorly understood. N^6^-methyladenosine (m^6^A) modifications are the most common form of reversible methylation observed on lncRNAs in eukaryotic cells, with their presence leading to pronounced changes in the activity of a range of biological processes. The degree to which m^6^A modification plays a role in the induction of apoptotic cell death in response to ionizing radiation (IR) in the context of RILI remains to be established. Here, IR-induced apoptosis was found to significantly decrease the levels of m^6^A present, with a pronounced decrease in the expression of methyltransferase-like 3 (METTL3) at 2 d post radiation in vitro. From a mechanistic perspective, a methylated RNA immunoprecipitation assay found that lncRNA MEG3 was a major METTL3 target. The expression of MEG3 was upregulated via METTL3-mediated m^6^A in a process that was dependent on YTHDC1, ultimately reversing the miR-20b-mediated inhibition of BNIP2 expression. Together, these findings demonstrate that the responsivity of METTL3 activity to IR plays a role in IR-induced apoptotic cell death, leading to the reverse of miR-20b-mediated BNIP2 inhibition through the YTHDC1-dependent m^6^A modification of MEG3, suggesting that this process may play a central role in RILI incidence.

## Introduction

The radiotherapeutic treatment of many tumors in the upper body or other regions in patients with certain cancers can result in the exposure of healthy hepatic tissue to ionizing radiation (IR), leading to the incidence of radiation-induced liver injury (RILI). In the liver, radiation can induce forms of disease such as non-icteric hepatomegaly and ascites that most often manifest within 2 weeks to 3 months post irradiation [1]. Following hepatic radiation at the lowest effective dose (30 Gy), the odds of RILI have been estimated at just 5% [2], yet they rise to >50% at a dose of 40 Gy, and 76% of liver cancer patients who receive a dose of 60 Gy will ultimately succumb to acute liver failure following treatment [3]. At present, the mechanistic basis for RILI has yet to be fully clarified, limiting any efforts to design effective interventional strategies. There is thus a pressing need to clarify the molecular etiology of RILI to guide its prevention and treatment.

RILI incidence is known to be closely associated with apoptotic cell death, oxidative stress, and fibrosis [4], with apoptosis occurring early during RILI [5]. The debris produced through extensive apoptotic cell death, together with inflammatory mediators and oxidative stress, are central to the induction of hepatic fibrosis [6].

**Fig. 1:**
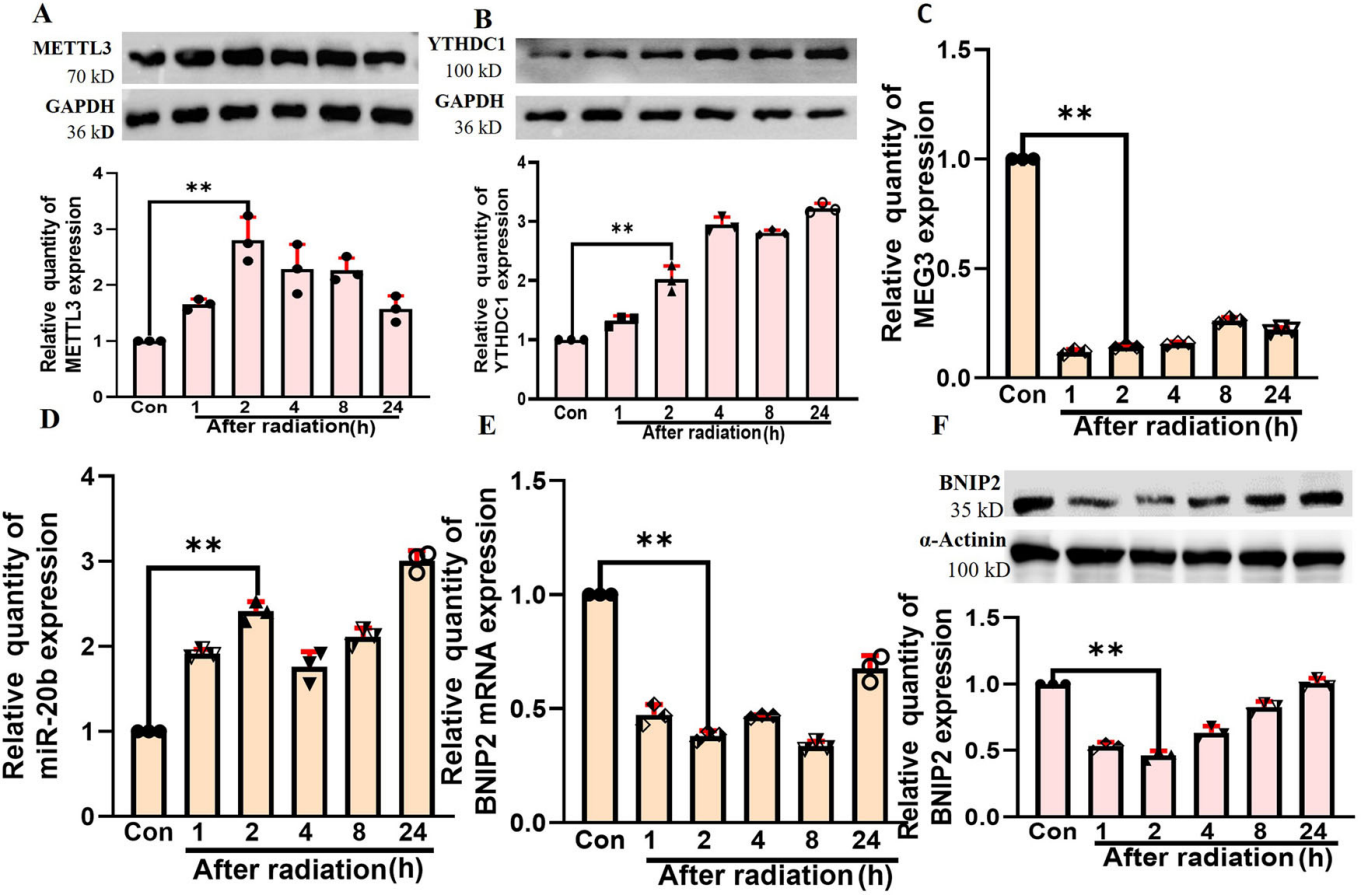
The impact of irradiation on METTL3, YTHDC1, MEG3, miR-20b, and BNIP2 levels in BNL CL2 cells. **A** The METTL3 protein level (**p < 0.01) at different time points within 24 h after radiation in BNL CL2 cells. **B** The YTHDC1 protein level (**p < 0.01) at different time points within 24 h after radiation in BNL CL2 cells. **C** The MEG3 level (**p < 0.01) at different time points within 24 h after radiation in BNL CL2 cells. **D** The miR-20b level (**p < 0.01) at different time points within 24 h after radiation in BNL CL2 cells. The BNIP2 mRNA (**E**) and protein (**F**) levels (**p < 0.01) at different time points within 24 h after radiation in BNL CL2 cells. **A**–**F** *n* = 3. **A**–**F** were followed by one-way ANOVA.

It was observed that MEG3 expression was dramatically downregulated in BNL CL2 cells within 24 h following radiation exposure (Fig. 1C). The binding proteins associated with MEG3 were predicted using the RBPDB database (http://rbpdb.ccbr.utoronto.ca/). Analysis revealed multiple binding sites between YTHDC1 and MEG3, suggesting potential methylation modifications of MEG3. Methylation sites of MEG3 were further predicted using the SRAMP tool (https://www.cuilab.cn/sramp). In vitro experiments demonstrated an increase in METTL3 levels post-cell radiation. Additionally, RNAhybrid version 2.2 was employed to predict the sponge targeting sites of MEG3 on miR-20b.

Much like mRNAs, long noncoding RNAs (lncRNAs) can undergo m^6^A modifications that can modulate their stability, translocation, transcriptional inhibition, and splicing [7, 8]. Methyltransferases (METTL3, METTL14), demethylases (FTO, ALKBH5), and reader proteins (YTHDC1-2, YTHDF1-3) are responsible for shaping the m^6^A modifications associated with target transcripts [9, 10]. While m^6^A methylation has been reported to be closely associated with apoptotic cell death [11], the functional role of m^6^A in the context of RILI pathogenesis remains uncertain, and the involvement of m^6^A methylation in the control of RILI-associated apoptosis has yet to be assessed.

Here, a significant increase in METTL3-mediated m^6^A modification levels was observed at 2 h post irradiation, followed 2 days later by a significant reduction in such activity. From a mechanistic perspective, METTL3 was determined to promote the m^6^A modification of the lncRNA MEG3, resulting in the recruitment of YTHDC1 and the degradation of MEG3, thereby enhancing the ability of miR-20b to inhibit BNIP2 expression. Treatment efforts focused on targeting this m^6^A modification axis mediated by METTL3 were sufficient to protect against RILI and associated IR-induced apoptotic death, suggesting that efforts to target this YTHDC1/ MEG3 axis may be a feasible means of protecting against RILI.

## Methods

### Materials

Murine embryonic liver cells (BNL CL2) and Human HEK293T cells were obtained from BNCC Biotech Inc. (BNCC 337873, 353535, Beijing, China). All cells were cultured in DMEM with 10% FBS, penicillin (100 U/mL), and streptomycin (100 U/mL) in a 5% CO_2_ incubator at 37 °C. Cell lines were cultured in standard conditions, routinely tested for mycoplasma contamination using BeyoDirect™ Mycoplasma qPCR Detection Kit (Beyotime Biotechnology Co., Ltd, Shanghai, China) and authenticated by STR profiling performed by using the PowerPlex® 18D System (Promega DC1802, USA) kit for gene amplification. Cells (1 × 10^6^ cells/mL) in logarithmic growth phase were seeded in plates. Male C57BL/6J mice from Beijing Wei-tong-li-Hua Experimental Animal Technology Co., Ltd. were housed in cages in a climate-controlled facility with a 12 h light/dark cycle. 6- to 8-week-old mice were randomly divided into four groups (*n* = 8). Investigators were blinded to the group allocation during the experiment. In vivo assays were performed as per the Guide for the Care and Use of Laboratory Animals with approval from the Animal Ethics Committee of Zaozhuang University (Shandong, China). BNIP2 antibody was purchased from Thermo Fisher Scientific Inc. (Waltham, MA, USA, PA5-101452). YTHDC1, METTL3, α-actinin and GAPDH were purchased from Proteintech Group Inc. (Wuhan, China, 14392-1-AP, 15073-1-AP, 11313-2-AP, 10494-1-AP). Caspase-3/cleaved antibody was purchased from Wan-Lei Biotechnology Co., Ltd (WL02117, Shenyang, China).

### Lentiviral injection

A YTHDC1-specific shRNA sequence (TGGATTTGCAGGCGTGAATTA, CGACCAGAAGATTATGATA) was used to establish the GV493 lentiviral vector (hU6-MCS-CBh-gcGFP-IRES-puromycin, GeneChem, Shanghai, China). The YTHDC1 and negative control (NC) shRNA plasmids were packaged in HEK293T cells with a plasmid ratio of pTarget:pVSVg:RRE:REV = 3:1:2:2. After 3 days, viruses secreted from these cells were harvested, centrifuged, filtered, and injected into mice via the tail vein. On days 1 and 4, animals were injected with a total volume of 100 µL. On day 7, samples of liver tissue were collected to assess YTHDC1 protein expression to confirm successful model establishment.

### Irradiation

Animals and cells were irradiated at room temperature with a total dose of 6 Gy being provided at an 800 cGy/min dose rate.

### Quantitative real-time PCR analysis

TRIzol (Invitrogen) was used for RNA isolation, followed by the analysis of these isolated samples with a Nanodrop 2000C (Thermo Scientific). Then, samples of RNA (2 µg) were reverse transcribed with ReverTra Ace qPCR RT Master Mix Kit (G3322, Wuhan Sevicebio Technology Co., Ltd., Wuhan, China) as directed, while a ring-based method was used to reverse transcribe miRNAs with miRNA RT primers. Thermocycler settings were: 95 °C for 1 min; 40 cycles of 95 °C for 15 s and 60 °C for 30 s; 72 °C for 45 s. Primers used for this study were produced by Wuhan Servicebio Biotechnology Co., Ltd (Wuhan, China) and are presented in Table 1.

**Table 1:** Oligonucleotides.

Gene	Primer
mmu-miR-20b-5p-RT	CTCAACTGGTGTCGTGGAGTCGGCAATTCAGTT GAGCTACCTGC
mmu-miR-20b-5p-S	ACACTCCAGCTGGGCAAAGTGCTCATAGTGC
M-BNIP2-S	TATTGGCACTTTAGAGCTGTTGG
M-BNIP2-A	AGTGTTCGGATAAACCAAGAGGG
M-Meg3-19-S	TTCAATGGGGCAATGGAGG
M-Meg3-19-A	TCCCATCCATTCATTTGTACTCTC

### MeRIP-qPCR analyses

A Ribo MeRIP^TM^ m6A Transcriptome Profiling Kit (R11096.6, Guangzhou Ruibo Biological Technology Co., Ltd. Guangzhou, China) was used to conduct MeRIP-qPCR analyses. Briefly, total RNA was isolated from transfected BNL CL2 cells that had been exposed to X-ray irradiation (6 Gy) at 2 h or 2 days post irradiation with TRIzol. Then, 500 μL of MeRIP reaction solution was combined with anti-m^6^A magnetic beads for 2 h at 4 °C, followed by the addition of 100 μL of elution buffer to samples after the IP reaction, mixing thoroughly to suspend samples. Samples were then shaken for 1 h at 4 °C, followed by the recovery of the eluted RNA captured with the m^6^A antibody. Both IP and input samples were analyzed via qPCR.

### RIP assay

RIP analyses were conducted using a BersinBio^TM^ RNA Immunoprecipitation (RIP) Kit (Bes5101, Guangzhou Bersinbio Co., Ltd. Guangzhou, China). After harvesting cells and lysing them in a lysis buffer supplemented with RNase and protease inhibitors, these lysates were incubated overnight at 4 °C with anti-YTHDC1 or IgG control. Next, 20 µl of prepared protein A/G magnetic beads were added to these samples followed by a further 1 h incubation at 4 °C. Polysome washing buffer was used to conduct five washes, and RNA attached to these beads was then isolated with TRIzol for subsequent qPCR analyses. When comparing RNA and protein binding, relative enrichment levels were first normalized to input, followed by comparisons with data from samples precipitated using anti-YTHDC1 and anti-IgG.

### RNA decay assay

RNA stability was evaluated by transfecting cells with YTHDC1-shRNA and then treating them using actinomycin D (10 μg/mL). After 0, 2, 4, or 8 h, cells were harvested and total RNA was isolated for qPCR-based analyses of relative MEG3 abundance [12].

### Statistical analysis

The data are presented as means ± standard deviation (SD) from all experiments. Statistical comparisons between two groups were conducted using a two-tailed Student’s T-test. For analyses involving three or more independent groups, one-way analysis of variance (ANOVA) followed by Tukey’s multiple comparison test was employed. The outcomes of *T*-test and one-way ANOVA test encompass the *F*-test, which assesses the homogeneity of variance. All statistical analyses were performed using GraphPad Prism version 8.2 (GraphPad Software, CA, USA), with a significance threshold set at *P* < 0.05. Details of number of replicates are provided in the individual figure legends.

## Results

### YTHDC1-associated m^6^A methylated proteins and MEG3-related molecules linked to apoptosis are induced following the irradiation of BNL CL2 cells

Predictive analyses of MEG3 identified a putative binding interaction with the target protein YTHDC1, indicating its m^6^A modification in the context of RILI. Further predictive analyses identified miR-20b as a MEG3 target miRNA, suggesting the ability of MEG3 to serve as a competing endogenous RNA to control BNIP2 expression. METTL3 and YTHDC1 upregulation was evident at 2 h post irradiation relative to the control group (Fig. 1A, B), whereas at later time points after irradiation MEG3 was downregulated (Fig. 1C). The upregulation of miR-20b was observed at 2 h post irradiation (Fig. 1D), while both BNIP2 mRNA and protein levels were reduced within 24 h post irradiation (Fig. 1E, F). Based on these findings, a 2 h time point was selected for downstream analyses.

## The m^6^A reader protein YTHDC1 influences BNL CL2 cell viability and apoptosis following radiation-induced injury

**Fig. 2:**
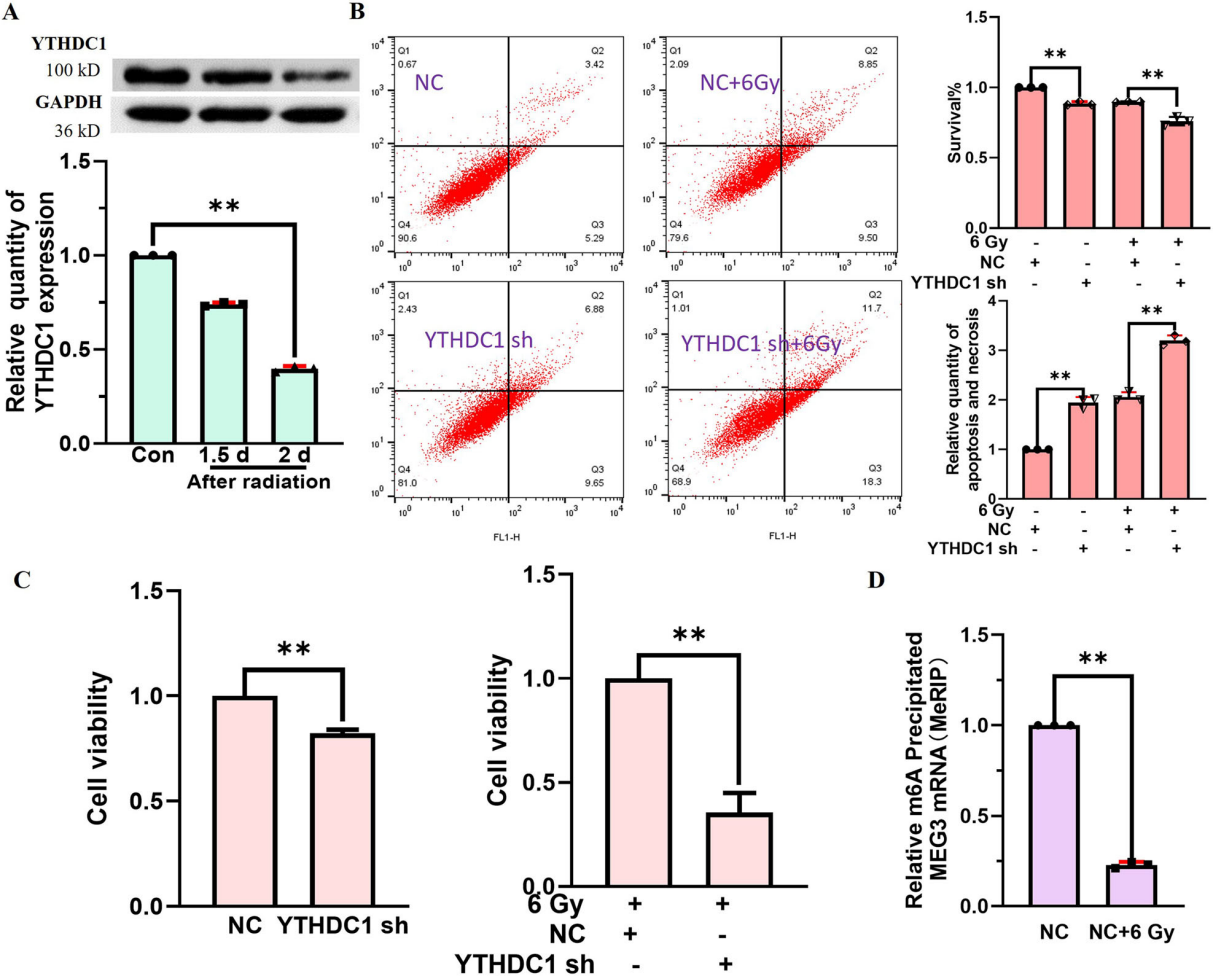
YTHDC1 impacts BNL CL2 cell viability and apoptotic induction following radiation-induced injury. **A** Western blotting revealed the downregulation of YTHDC1 in BNL CL2 cells on day 2 post irradiation (**P < 0.01). **B** Flow cytometry was used to examine the impact of YTHDC1 on BNL CL2 cell apoptosis on day 2 post irradiation (**P < 0.01). C. A CCK-8 assay was used to examine the impact of YTHDC1 on BNL CL2 cell viability with or without irradiation (**P < 0.01). D. The methylation level of MEG3 in BNL CL2 cells a at 2 d after 6 Gy radiation was detected by MeRIP assay (**P < 0.01). A, B, D, n = 3; C, *n* = 5. **A**, **B** were followed by one-way ANOVA. **C**, **D** were performed by two-tailed unpaired T-test.

To better understand how YTHDC1 affects post-irradiation apoptotic cell death, YTHDC1 levels were analyzed 1.5 and 2 days after irradiation. At 24 h after irradiation, elevated YTHDC1 levels were evident in BNL CL2 cells(Fig. 1C), whereas it was downregulated significantly at 48 h after irradiation (Fig. 2A). Flow cytometry analyses revealed that the knockdown of YTHDC1 increased apoptotic induction, with higher apoptosis rates in the YTHDC1-knockdown group relative to the NC (negative control) group on day 2 post irradiation (Fig. 2B). When an shRNA vector was used to knockdown YTHDC1, these cells exhibited greater radiation sensitivity as compared to shRNA control cells, with a significant reduction in cellular viability in the irradiated YTHDC1-knockdown group as compared to NC irradiated cells (Fig. 2C). The methylation level of MEG3 detected by MeRIP assay in BNL CL2 cells a at 2 h after 6 Gy radiation was downregulated (Fig. 2D). These findings support that the ability of YTHDC1 to regulate the viability and apoptosis of BNL CL2 cells after irradiation may be related to MEG3 methylation.

### MEG3 influences BNL CL2 cell viability and apoptosis following radiation-induced injury

**Fig. 3:**
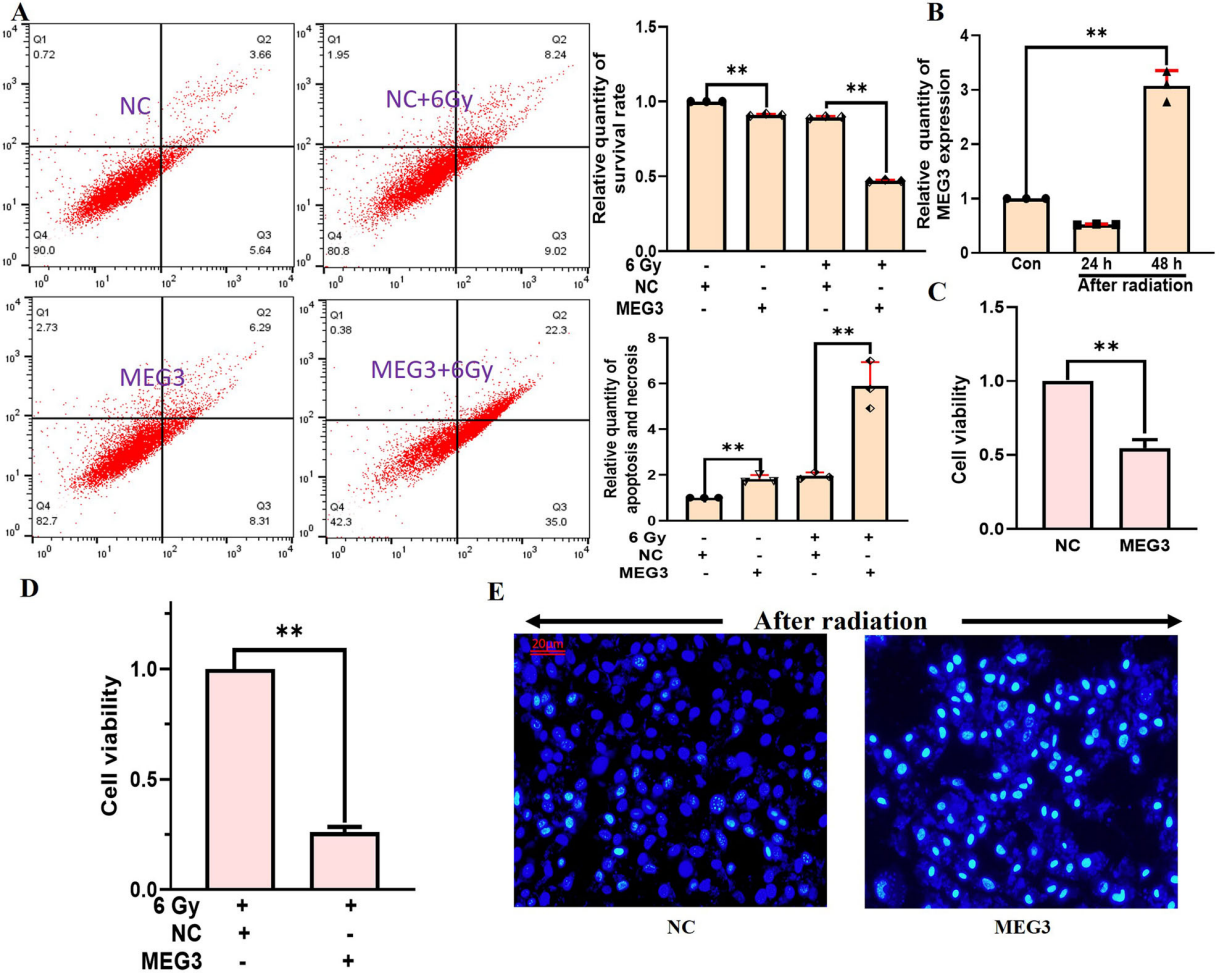
MEG3 influences BNL CL2 cell viability and apoptosis following radiation-induced injury. **A** Flow cytometry was used to assess the effects of MEG3 on BNL CL2 cell apoptosis on day 2 post irradiation (**P < 0.01). B. MEG3 was significantly upregulated on day 2 post irradiation (**P < 0.01). **C** A CCK-8 assay was used to assess the impact of MEG3 on BNL CL2 cell viability (**P < 0.01). **D** A CCK-8 assay was used to assess the impact of MEG3 on BNL CL2 cell viability on day 2 post irradiation (**P < 0.01). **E** Hoechst staining was used to examine the impact of MEG3 on BNL CL2 cell apoptosis on day 2 post irradiation. **A**, **B** *n* = 3; **C**, **D** *n* = 5; scale bar: 20 µm. **A**, **B** were followed by one-way ANOVA. **C**, **D** were performed by two-tailed unpaired T-test.

To test the impact of MEG3 on post-irradiation apoptosis, this lncRNA was overexpressed. Such MEG3 overexpression increased the rates of cell apoptosis in the absence of irradiation and on day 2 post irradiation (Fig. 3A). Significant MEG3 upregulation was confirmed by qPCR on day 2 post irradiation (Fig. 3B). Cells overexpressing MEG3 exhibited greater radiation sensitivity and significantly reduced viability as compared to NC irradiated cells (Fig. 3C, D). Hoechst staining confirmed higher rates of MEG3-overexpressing cell apoptosis on day 2 post irradiation relative to the radiation-only group (Fig. 3E). These results supported the ability of MEG3 to induce apoptosis and to reduce the viability of irradiated BNL CL2 cells.

### METTL3-mediated m^6^A methylation of MEG3 influences radiation-induced hepatocyte injury

**Fig. 4:**
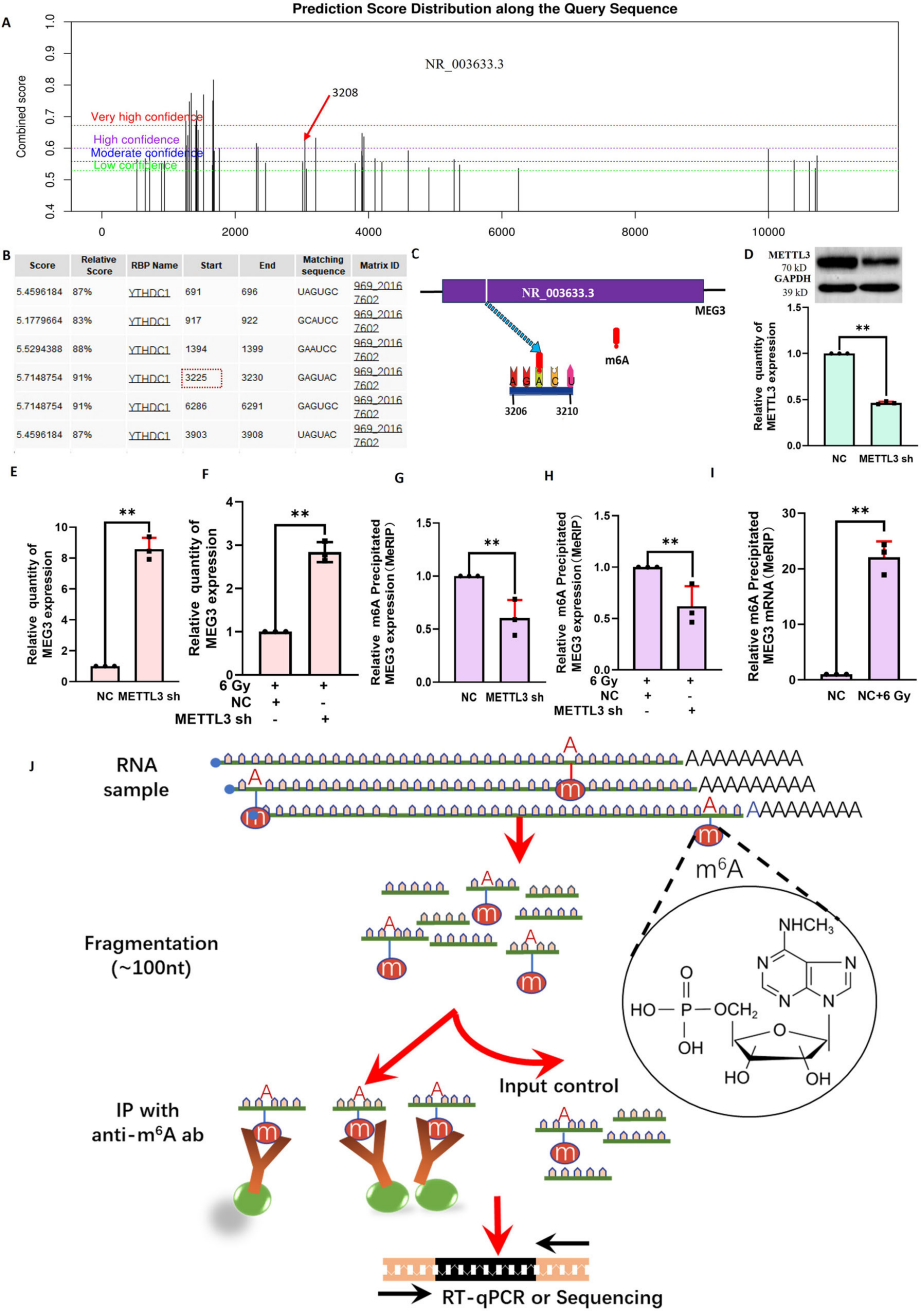
The m^6^A methylation of MEG3 mediated by METTL3 plays a role in radiation-induced hepatocyte injury. **A** Predictive analysis of MEG3-m^6^A methylation sites. **B** Predictive analysis of putative binding sites between MEG3 and YTHDC1. **C** Through the combination of results from (**A**, **B**), YTHDC1-dependent methylation sites were identified. **D** Confirmation of the successful knockdown of METTL3 in BNL CL2 cells (**P < 0.01). qPCR was used to detect levels of MEG3 in cells following METTL3 knockdown without (**E**) or with (**F**) 6 Gy irradiation (**P < 0.01). MeRIP was used to detect MEG3 methylation levels in BNL CL2 cells in which METTL3 was knocked down without (**G**) or with (**H**) 6 Gy irradiation (**P < 0.01). **I** MEG3 methylation levels were detected via MeRIP assay in BNL CL2 cells at 2 h following 6 Gy irradiation (**P < 0.01). **J** Overview of the MeRIP experimental approach. **D**–**I** *n* = 3. **D**–**I** were performed by two-tailed unpaired *T*-test.

Given the clear roles that YTHDC1 and MEG3 play in the pathogenesis of RILI in vitro, the ability of METTL3 to mediate MEG3 m^6^A modification was next explored at length. A predictive tool (https://www.cuilab.cn/sramp) was used to identify MEG3 methylation modification sites (Fig. 4A), while a separate database (http://rbpdb.ccbr.utoronto.ca/) identified multiple target binding sites in MEG3 and YTHDC1. The ability of MEG3 and YTHDC1 to interact was initially assessed (3225) (Fig. 4B), and the combination of these results led to the identification of a YTHDC1-dependent methylation modification site (3208) (Fig. 4C). Knockdown of METTL3 in BNL CL2 cells was achieved via lentivirus-mediated shRNA delivery (Fig. 4D), and MEG3 levels were found to be elevated following such METTL3 silencing in the presence or absence of irradiation (Fig. 4E, F). After knocking down METTL3 in BNL CL2 cells, MeRIP assays revealed significant reductions in m^6^A modification levels in the presence or absence of irradiation (Fig. 4G, H). At 2 h post irradiation, MeRIP assays indicated that MEG3 methylation modification levels were increased (Fig. 4I). An overview of the utilized MeRIP assay approach is shown in Fig. 4J. These results indicated that METTL3-mediated m^6^A modification can drive the degradation of MEG3, with this process playing a role in hepatocyte injury upon irradiation.

### METTL3 controls m^6^A-modified MEG3 expression in a manner dependent on YTHDC1 to regulate radiation-induced hepatocyte injury

**Fig. 5:**
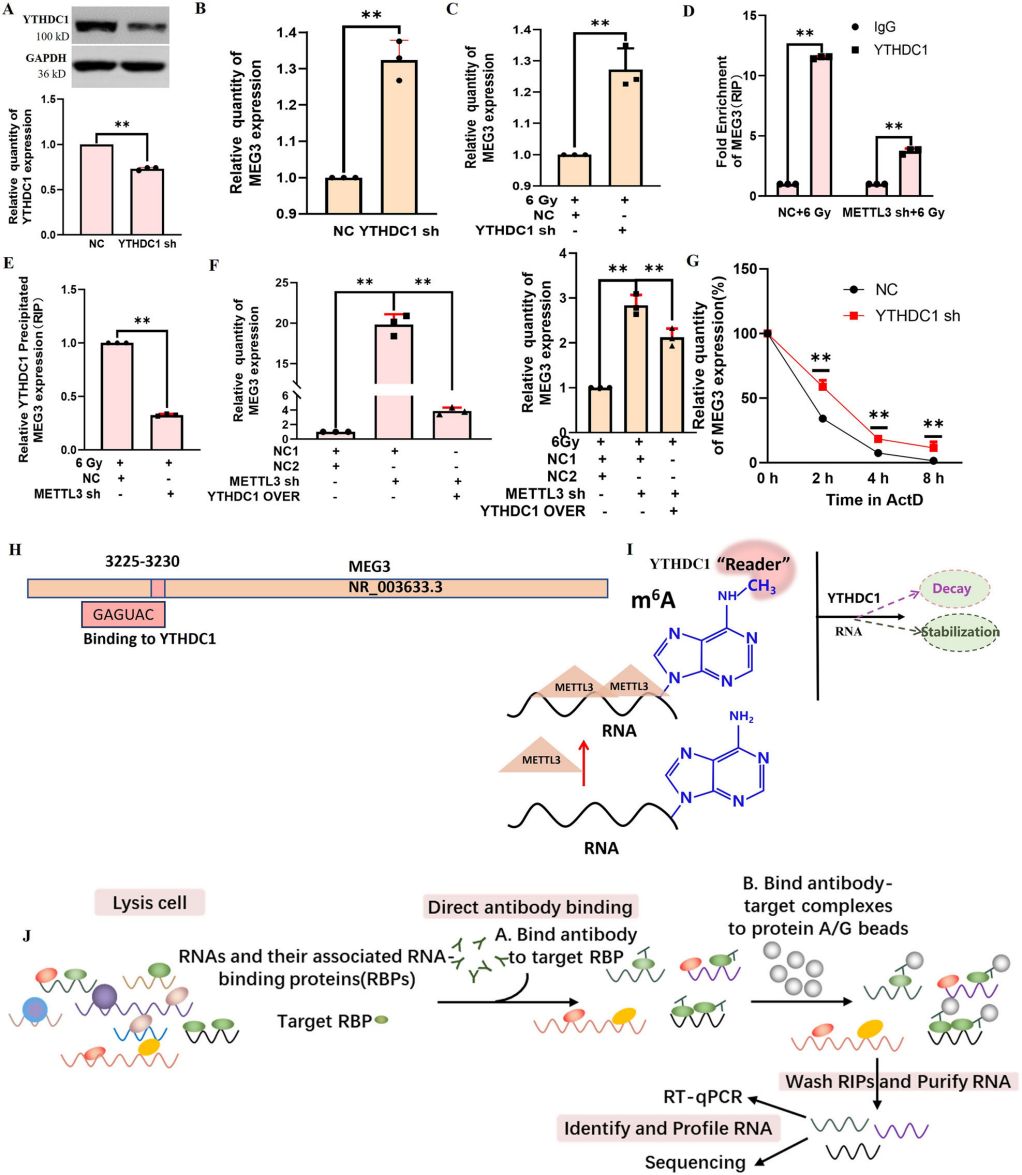
METTL3 controls MEG3 levels in a YTDC1-dependent fashion in the context of RILI. **A** Confirmation of the establishment of YTHDC1-knockdown BNL CL2 cells (**P < 0.01). **B** MEG3 expression in BNL CL2 cells was analyzed via qPCR in YTHDC1 knockdown cells (**P < 0.01). C. MEG3 expression levels were analyzed via qPCR following YTHDC1 knockdown and irradiation in BNL CL2 cells (**P < 0.01). **D**, **E** RIP assays were used to pull down MEG3 with anti-YTHDC1 in BNL CL2 cells following METTL3 knockdown and irradiation (**P < 0.01). **F** MEG3 levels were assessed in cells in which METTL3 had been knocked down and co-transfected with TYHDC1 with or without irradiation (**P < 0.01). **G** MEG3 levels were detected in NC or YTHDC1 shRNA-treated cells by qPCR following actinomycin D (1 µg/ml) treatment for the indicated periods (**P < 0.01). **H** MEG3 and YTHDC1 target binding sites. **I** YTHDC1 can recognize METTL3-mediated MEG3-m^6^A modification. **J** RIP experimental overview. **A**–**G**, *n* = 3. **A**–**G** were performed by two-tailed unpaired *T*-test.

Next, experiments were performed to test the ability of YTHDC1 to recognize METTL3-mediated m^6^A methylation. A lentiviral approach was used to knockdown YTHDC1 in BNL CL2 cells (Fig. 5A), resulting in the upregulation of MEG3 in these cells in the absence or presence of irradiation (Fig. 5B, C). At 2 h post irradiation in METTL3 knockdown cells, a reduction in MEG3 precipitation by YTHDC1 was observed in a RIP assay (Fig. 5D, E). When YTHDC1 was overexpressed in METTL3 knockdown cells, this approach reversed the upregulation of MEG3 induced by knocking down METTL3 (Fig. 5F). RNA was isolated from YTHDC1-knockdown cells at 0, 2, 4, and 8 h following the addition of actinomycin D, revealing lower MEG3 levels in the NC group relative to the YTHDC1-knockdown group, thus demonstrating that YTHDC1 promotes MEG3 degradation (Fig. 5G). MEG3 and YTHDC1 target binding sites are presented in Fig. 5H. The mechanism through which YTHDC1 recognizes METTL3-mediate MEG3 m^6^A modifications is presented in Fig. 5I. An overview of the utilized RIP approach is presented in Fig. 5J. In summary, METTL3-mediated m^6^A methylation can promote MEG3 degradation in a YTHDC1 dependent manner after radioactive hepatocyte injury.in a YTHDC1-dependent fashion in the context of radiation-induced hepatocyte injury.

### MEG3 targets miR-20b to upregulate BNIP2 in BNL CL2 cells, thus shaping radiation-associated damage

**Fig. 6:**
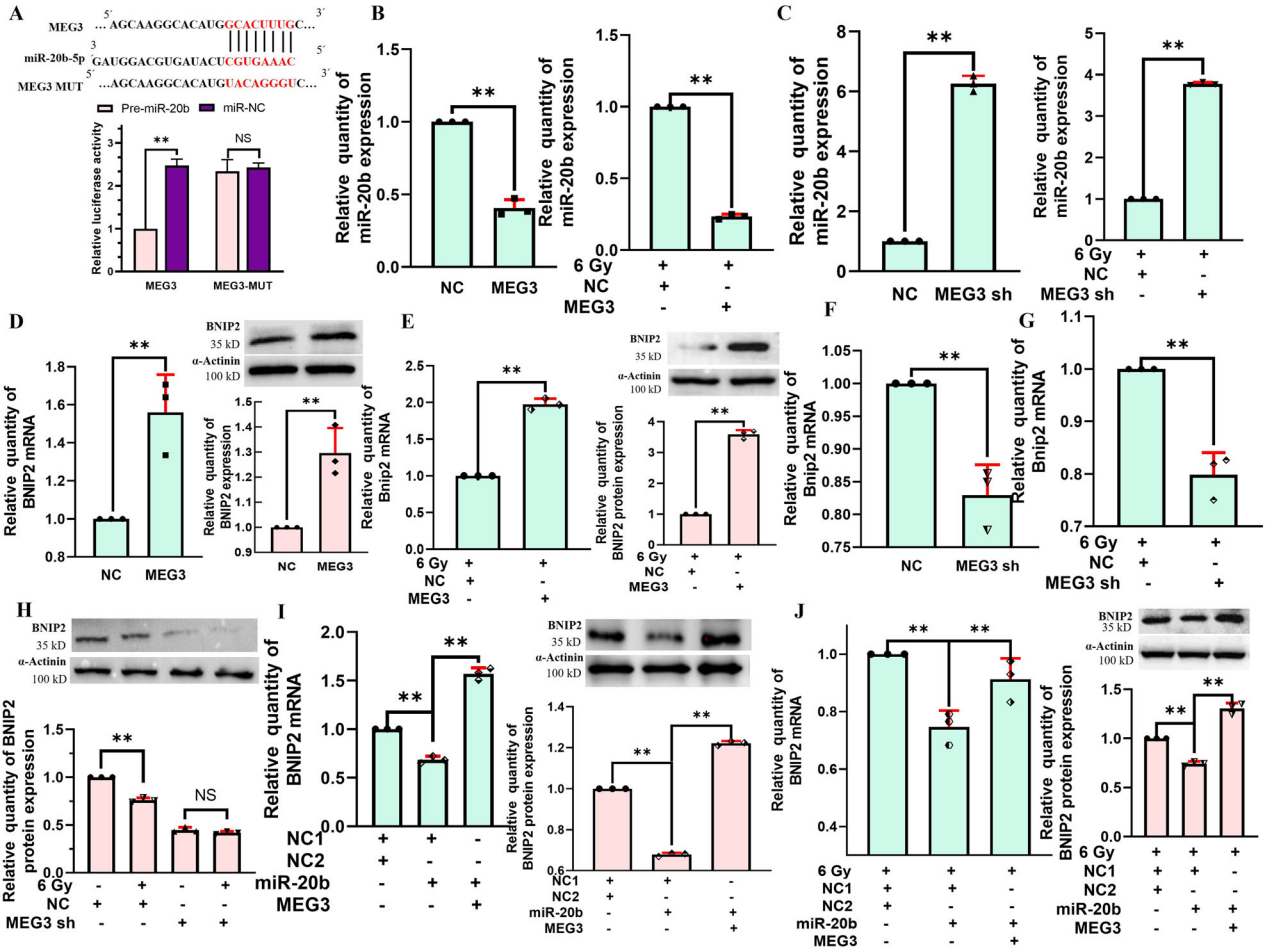
MEG3 regulates radiation-induced injury in BNL CL2 cells via regulating the miR-20b-mediated control of BNIP2 expression. **A** A dual-luciferase reporter assay was used to probe the impact of miR-20b on wild-type or mutant MEG3 reporter activity (**P < 0.01; NS no significance); The effects of irradiation (6 Gy) on miR-20b levels were assessed following the overexpression (**B**) or knockdown (**C**) of MEG3 (**P < 0.01). The impact of overexpressing MEG3 on BNIP2 mRNA and protein levels without (**D**) or with (**E**) irradiation (**P < 0.01). The impact of knocking down MEG3 on BNIP2 mRNA levels without (**F**) or with (**G**) irradiation. **H** The impact of knocking down MEG3 on BNIP2 protein levels in the presence or absence of irradiation (**P < 0.01, NS, no significance); MEG3 is capable of reversing miR-20b-mediated BNIP2 downregulation in the absence (**I**) or presence (**J**) of irradiation (**P < 0.01). **B**–**J** *n* = 3. **B**–**G** were performed by two-tailed unpaired *T*-test. **H**–**J** were followed by one-way ANOVA.

The competing endogenous RNA (ceRNA) relationships among MEG3, miR-20b, and BNIP2 were next explored in greater detail. Sites for the adsorption of miR-20b-5p by MEG3 were predicted using RNAhybrid 2.2, and a dual-luciferase reporter assay confirmed significant reductions in luciferase activity in cells transfected with the wild-type MEG3 reporter and miR-20b relative to that in cells transfected with the wild-type MEG3 reporter and miR-NC. In contrast, no significant differences between the miR-20b and miR-NC groups were evident when co-transfected with a mutated version of the MEG3 reporter vector, thus confirming the ability of miR-20b to directly target MEG3. MEG3 can thus function as a molecular sponge to adsorb miR-20b (Fig. 6A). Relative to the NC group, miR-20b levels in the MEG3 group were significantly reduced in the presence or absence of irradiation (Fig. 6B), while the opposite was observed following MEG3 silencing (Fig. 6C), offering further support for the status of miR-20b as a MEG3 target miRNA. BNIP2 mRNA and protein levels were significantly elevated in the MEG3 group in the presence or absence of irradiation (Fig. 6D, E), while knocking down MEG3 had the opposite effect (Fig. 6F–H). In the presence or absence of irradiation, MEG3 was able to reverse miR-20b-mediated decreases in BNIP2 levels, suggesting the miR-20b-dependence of this MEG3-driven upregulation of BNIP2 (Fig. 6I, J). Overall, these findings are consistent with a role for MEG3 as a regulator of BNL CL2 cell radiation-induced damage through its ability to bind miR-20b and upregulate BNIP2.

### miR-20b protects against radiation-induced BNL CL2 cell injury through the targeted downregulation of BNIP2

**Fig. 7:**
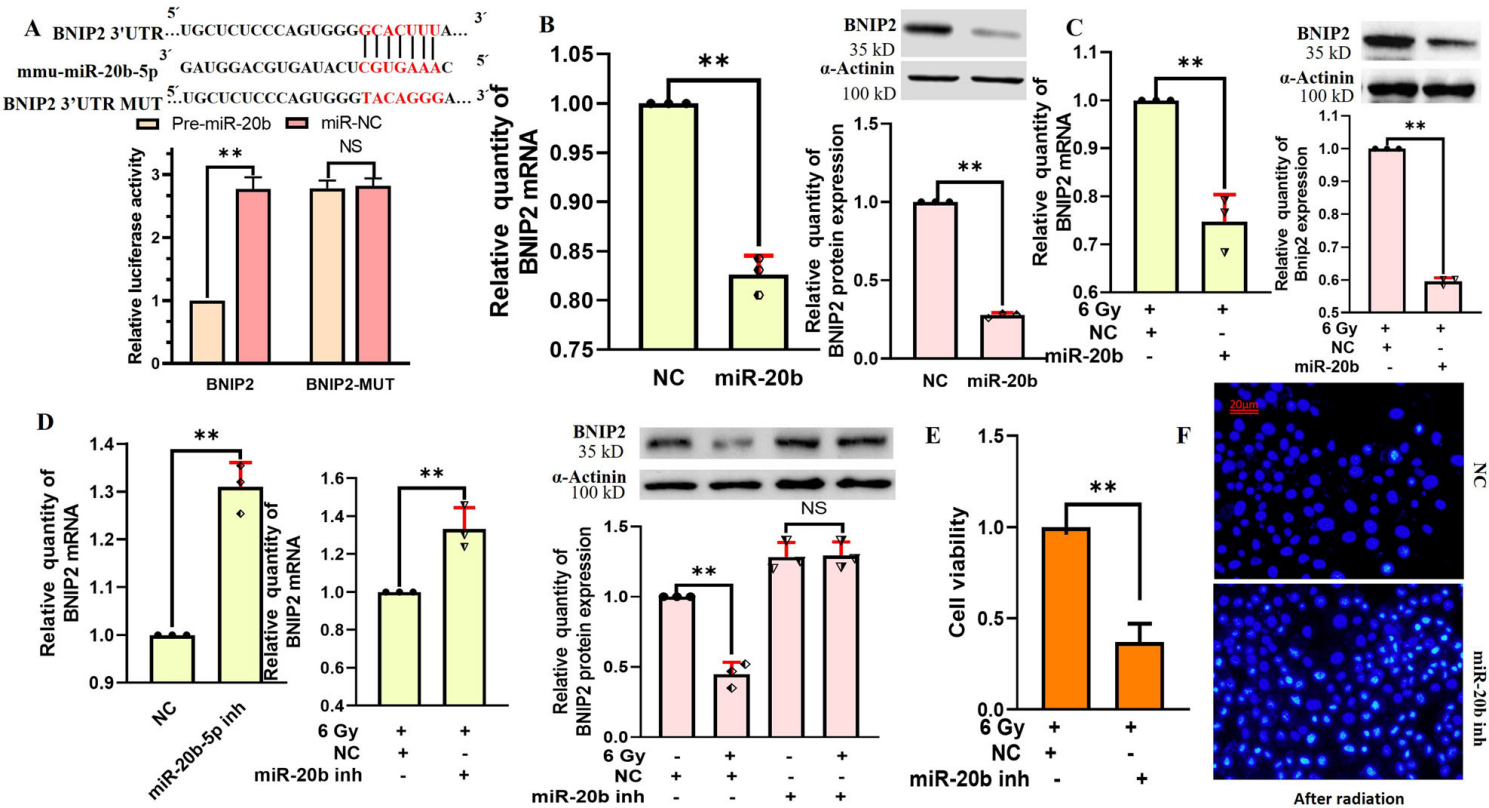
miR-20b protects against radiation-induced injury in BNL CL2 cells through targeting BNIP2. **A** A dual-luciferase reporter assay was used to probe the impact of miR-20b on the activity of wild-type or mutant BNIP2 (**P < 0.01; NS, no significance). The impact of overexpressing miR-20b on BNIP2 mRNA and protein levels within BNL CL2 cells without (**B**) or with (**C**) irradiation. (**P < 0.01). **D** The impact of knocking down miR-20b on the mRNA and protein levels of BNIP2 in BNL CL2 cells that were or were not irradiated (**P < 0.01, NS no significance). **E** A CCK-8 assay was used to assess the effects of miR-20b inhibition on BNL CL2 cell viability following miR-20b inhibition and irradiation (**P < 0.01). **F** Hoechst staining was used to assess the impact of miR-20b inhibition on BNL CL2 cell apoptosis following irradiation. **B**–**D**, *n* = 3; **A**, **E**, *n* = 5; scale bar: 20 µm. **B**–**E** were performed by two-tailed unpaired *T*-test. **D** was also followed by one-way ANOVA.

TargetScan was utilized to predict the potential of BNIP2 as a target of miR-20b. In a luciferase reporter assay performed with HEK293T cells, miR-20b significantly reduced reporter activity in cells co-transfected with a reporter containing the wild-type version of the predicted BNIP2 relative to miR-NC. In contrast, when this binding sequence was mutated, luciferase activity was comparable in the miR-20b and miR-NC groups (Fig. 7A), confirming the ability of miR-20b to directly target BNIP2. Relative to the NC group, BNIP2 mRNA and protein levels were reduced in the miR-20b treatment group (Fig. 7B), and miR-20b retained its ability to reduce these BNIP2 mRNA and protein levels following irradiation (Fig. 7C). Relative to the NC group, miR-20b inhibition increased BNIP2 mRNA and protein levels irrespective of irradiation status (Fig. 7D). When cell viability was analyzed at 2 days post irradiation following the knockdown of miR-20b, cells in which miR-20b expression was impaired exhibited greater radiation sensitivity (Fig. 7E). Following miR-20b knockdown, higher levels of apoptosis measured via Hoechst staining were observed at 2 days post irradiation relative to control irradiated cells (Fig. 7F). These findings suggest the ability of miR-20b to target BNIP2 and thereby disrupt radiation-induced BNL CL2 injury.

### YTHDC1 recognizes m^6^A-modified MEG3 to influence the pathogenesis of RILI in vivo

**Fig. 8:**
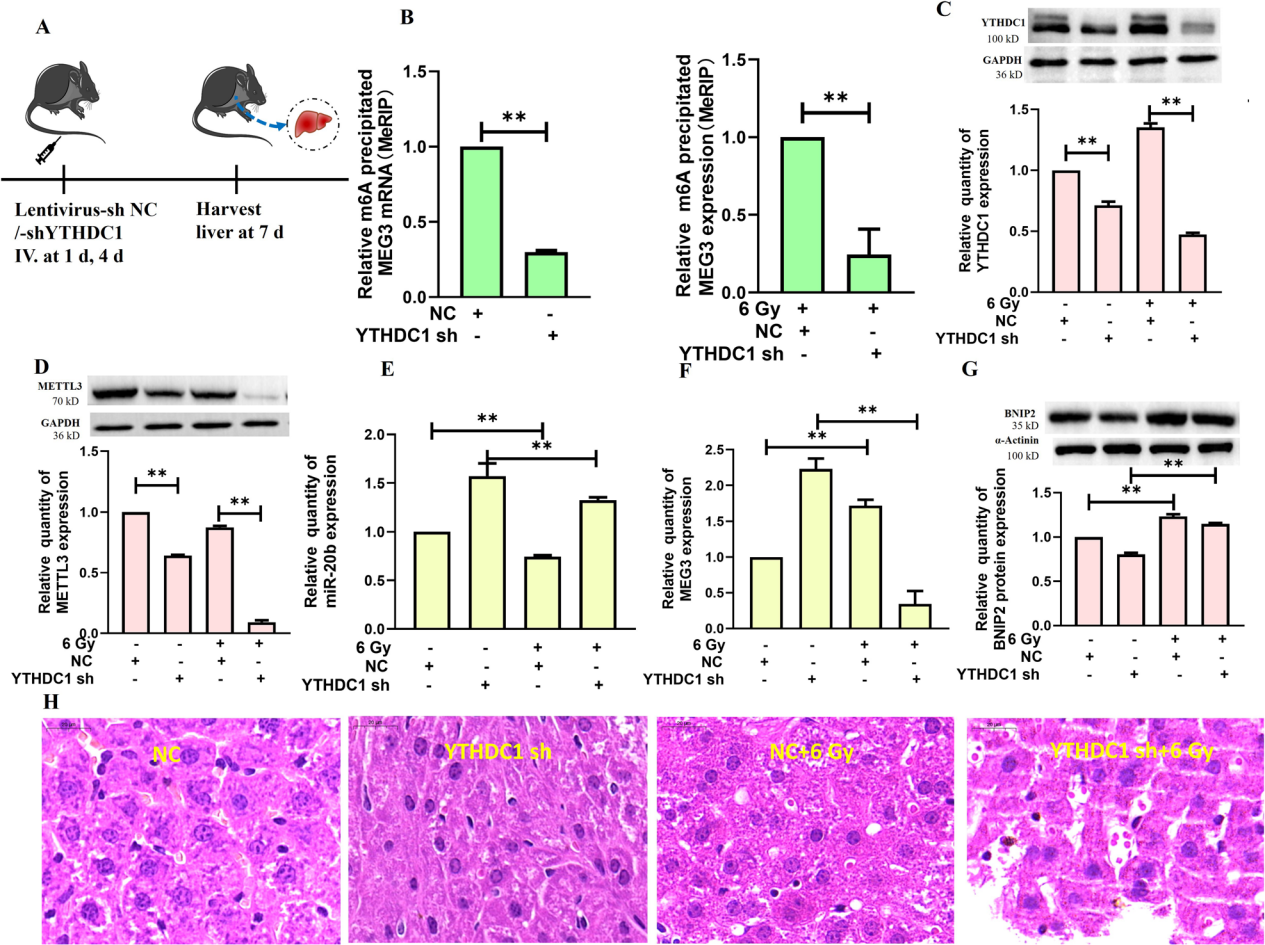
YTHDC1 plays a role in RILI in mice through the identification of m^6^A-modified MEG3. **A** Schematic overview of the preparation of a YTHDC1-knockdown mouse model. **B** Hepatic MEG3 methylation modification levels were measured following YTHDC1 knockdown in mice without or with radiation (**P < 0.01). **C** The impact of knocking down YTHDC1 on hepatic YTHDC1 protein levels was assessed in mice that were or were not irradiated (**P < 0.01) **D** The impact of silencing YTHDC1 on the hepatic protein levels of METTL3 was assessed in mice that were or were not irradiated (**P < 0.01); E. The impact of silencing YTHDC1 on the hepatic levels of miR-20b was assessed in mice that were or were not irradiated (**P < 0.01); **F** The impact of silencing YTHDC1 on the hepatic MEG3 levels was assessed in mice that were or were not irradiated (**P < 0.01); **G** The effects of silencing YTHDC1 on BNIP2 protein in the liver of mice that were or were not irradiated (**P < 0.01); **H** Pathological changes in murine liver tissue were assessed via H&E staining following the knockdown of YTHDC1 and irradiation-induced damage. **B**–**G** *n* = 8; scale bar: 20 µm. **B** was performed by two-tailed unpaired *T*-test. **C**–**G** were followed by one-way ANOVA.

YTHDC1 was knocked down in mice to explore its relationship with MEG3 in the context of RILI (Fig. 8A). When hepatic samples were collected from mice in the NC and YTHDC1 knockdown groups, lysates were analyzed via MeRIP-qPCR to detect MEG3 levels. Relative to the NC group, significantly lower MEG3 precipitated by m6A was observed in the YTHDC1 knockdown group without or with radiation (Fig. 8B). Relative to the NC group, significantly lower METTL3 and YTHDC1 protein levels were also observed in the YTHDC1 knockdown group (Fig. 8C, D) irrespective of irradiation status. Following irradiation, a reduction in miR-20b levels was detected in both the NC and YTHDC1sh groups of mice (Fig. 8E). Notably, MEG3 expression was upregulated in irradiated NC mice, whereas it was downregulated in irradiated YTHDC1-knockdown mice (Fig. 8F). Furthermore, an increase in BNIP2 levels was observed across all irradiated groups, irrespective of YTHDC1 knockdown status (Fig. 8G). Following irradiation, there was a significant increase in the levels of cleaved caspase-3 (17kD) proteins in both the NC and YTHDC1 shRNA groups. However, the magnitude of this increase was less pronounced in the YTHDC1 shRNA group compared to the NC group (S-Fig. 1). Compared to the NC group, the NC + 6 Gy group exhibited significantly elevated levels of AST, ALT, LDH, and ALP. In contrast, the YTHDC1 shRNA+6 Gy group demonstrated significant reductions in AST, ALT, and LDH levels relative to the YTHDC1 shRNA group, although the ALP level was significantly increased. Additionally, the NC + 6 Gy group showed significantly higher TP, ALB, and GLB values compared to the NC group (S-Fig. 2). The NC and YTHDC1-knockdown mice were used to establish a RILI model system, with liver tissues from these mice being harvested for H&E staining. Relative to NC controls, YTHDC1-knockdown mice exhibited reduced local integrity, chromatin agglutination/fragmentation, new distribution around the nuclear membrane, and cytoplasmic shrinkage. As compared to the irradiated NC mice (6 Gy), the irradiated YTHDC1 mice exhibited larger cellular spaces, chromatin condensation, nuclear dissolution, and the enlargement of hepato-blood sinuses (Fig. 8H). The TUNEL staining results indicated that, compared to the NC group, the hepatocyte apoptosis levels were significantly elevated in the NC + 6 Gy group of mice. The knockdown of hepatic YTHDC1 resulted in a further increase in hepatocyte apoptosis. Moreover, when the combined effects of YTHDC1 knockdown and radiation exposure were assessed (YTHDC1 shRNA+6 Gy group), there was an even more pronounced increase in hepatocyte apoptosis compared to the levels observed in the NC + 6 Gy group subjected to radiation alone (S-Fig. 3). These results support the ability of YTDC1 to modulate the pathogenesis of RILI through the regulation of MEG3 expression within murine liver tissue. These results indicated that YTDC1 could regulate the methylation of MEG3 in mouse liver tissue, and miR-20b targeted and negatively regulated BNIP2, thereby influencing the pathogenesis of RILI.

## Discussion

Multiple studies have documented important roles for m^6^A modifications in a range of physiological settings and diseases [13, 14]. Here, a positive correlation between m^6^A modification and IR-induced apoptotic death was detected, underscoring the ability of this alteration to shape RILI pathogenesis. Specifically, IR exposure was found to induce significant changes in m^6^A modification patterns and apoptotic cell death early during the onset of RILI in vitro and in vivo. Suppressing the effect of this m^6^A modification through the knockdown of YTHDC1 was sufficient to enhance IR-driven apoptotic death, while luciferase reporter assays and MeRIP-qPCR were employed to confirm interactions between METTL3 and MEG3. Together, these data clearly reveal the ability of the METTL3-mediated m^6^A modification of MEG3 to recruit YTHDC1, reducing its expression and thereby resulting in enhanced miR-20b-mediated inhibition of BNIP2.

Radiation can directly impact the structure of RNA, thereby affecting m^6^A methyltransferase binding and catalytic activity, potentially resulting in marked reductions in m^6^A methylation levels [15]. Several studies have offered support for the ability of m^6^A to influence gene expression, ultimately controlling a range of processes including self-renewal, differentiation, apoptosis, and invasivity [16]. METTL3 can suppress autophagic flux and enhance the induction of apoptotic death in cardiomyocytes subjected to hypoxia/reoxygenation through the m^6^A modification of TFEB and the consequent inhibition of the expression of the key regulator of autophagy-related genes and lysosomal biogenesis [17]. Knocking down METTL3 can significantly lower m^6^A levels, interfere with cellular growth, and increase the rate of apoptotic death [18]. Here, a previously unreported role for m^6^A modification in the regulation of RILI was reported. Increases in m^6^A levels mediated by the upregulation of METTL3 were observed in the context of RILI and found to be closely associated with IR-induced adaptive protection potentially at 2 h post irradiation. Decreases in these m^6^A levels during the course of RILI were found to be closely associated with IR-induced apoptotic death, potentially as a result of the downregulation of METTL3 on day 2 post irradiation. These findings support a possible role for METTL3-mediated m^6^A modification in the pathogenesis of RILI. MEG3 was identified as a major target of m^6^A modification mediated by METTL3 upon irradiation during RILI. MeRIP-qPCR was used to confirm the ability of METTL3 to target MEG3 to undergo m^6^A modification, and luciferase reporter assays further confirmed this relationship.

RNA-seq analyses indicated that IR treatment reduced MEG3 expression levels, and subsequent predictive analyses identified YTHDC1 as a binding partner for this lncRNA. YTHDC1 is capable of binding to methylated RNA, forming transcriptional aggregates via multivalent interactions with transcriptional activators capable of promoting the activation of transcription. YTHDC1 can additionally bind to RNAs to inhibit transcription or to alter the stability of RNAs subjected to m^6^A modification [19]. YTHDC1 shRNA has been shown to increase rates of keratinocyte apoptosis and to impair wound healing activity [20]. YTHDC1 silencing can suppress bile duct cancer cell proliferative, migratory, and invasive activity, lead to G2/M cell cycle arrest, and promote apoptotic death [21]. Enriched YTHDC1 was detected on MEG3, and knocking down YTHDC1 increased MEG3 levels within BNL CL2 cells that had been irradiated (RIP). Together, these data support the status of MEG3 as a direct YTHDC1 target.

LncRNAs are vital regulators of radiation-induced damage, cell growth, differentiation, oncogenic progression, and apoptosis [22–25]. The 1.6 kb lncRNA MEG3 is encoded on chromosome 14q32.3 in humans [26, 27]. Prior reports have established the tumor suppressor-like effects of MEG3 in cancers including glioma, gastric cancer, and melanoma [28, 29]. MEGα a MEG3 subtype that can inhibit cell growth, leading some researchers to posit that MEG3 is an important regulator of cellular proliferation [30]. At 48 h following whole-body irradiation, MEG3 upregulation was detected in murine blood [31]. MEG3 expression has also been shown to activate the pyroptosis of neurons through the NLRP3/caspase-1/GSDMD signaling pathway in a m^6^A-dependent fashion, thereby resulting in ischemic brain injury [32]. MEG3 m^6^A modification also facilitated apoptotic cell death, oxidative stress, and mitochondrial dysfunction induced by oxygen-glucose deprivation/reperfusion treatment in HT-22 cells [33]. Here, MEG3 was established as an important target of m^6^A mediated by METTL3 in the context of RILI. MeRIP-qPCR was initially used to confirm the ability of METTL3 to target MEG3 to undergo m^6^A modification. Decreased MEG3 levels were observed at 2 h post irradiation, with these decreases being eliminated when METTL3 was knocked down. Luciferase reporter assays also confirmed the interaction between METTL3 and MEG3 in the context of m^6^A modification.

Through their ability to serve as molecular sponges, lncRNAs have been shown to suppress the activity levels of a range of miRNAs, leading to the posttranscriptional derepression of miRNA targets [34]. Many cancers present with miR-20b upregulation, and it can serve as an oncogenic mediator linked to poor prognostic outcomes in these diseases [35, 36]. Specifically, miR-20b-5p promotes enhanced proliferative, migratory, and invasive activity together with reduced apoptosis [37]. This study is the first report demonstrating the ability of MEG3 to target miR-20b. Another Bcl-2 family proteins regulate apoptosis, and BNIP2/NIP2 is a unique member of this family that harbors a BCH domain (BNip2 and Cdc42GAP Homology) rather than the conserved BH3 domain. The BNIP2 BCH domain is essential for apoptotic cell death, as evidenced by previous studies [38, 39]. BNIP2, a Bcl-2 homology domain-only protein within the Bcl-2 family, is integral to mitochondrion-mediated apoptosis [40]. Furthermore, BNIP2 has been implicated in apoptosis induced by the 1-methyl-4-phenylpyridinium ion [41]. Similarly, BNIP2 has been identified as a proapoptotic factor in the context of estrogen-mediated neuroprotection [42]. This is the first report identifying BNIP2 as a miR-20b target, with the ability of miR-20b to inhibit BNIP2 being attenuated by the overexpression of MEG3 in the context of RILI.

In comparison to the NC group, the NC + 6 Gy group exhibited a significant increase in the levels of AST, ALT, LDH, and ALP [43]. This elevation can be attributed to hepatocyte damage, which causes these cytoplasmic indicators to be released into the bloodstream, thereby increasing their corresponding serum levels. At an advanced stage of liver injury, characterized by extensive hepatocyte death and substantial impairment of their functions, the YTHDC1 shRNA+6 Gy group exhibited a notable reduction in the release of AST, ALT, and LDH compared to the YTHDC1 shRNA group, resulting in significantly decreased serum levels of these enzymes in the YTHDC1 shRNA+6 Gy group, although the ALP levels continued to rise. In comparison to the NC group, the NC + 6 Gy group also exhibited a significant increase in the levels of TP, ALB, and GLB. This elevation can be attributed to the impairment of hepatic synthetic function, which subsequently initiates compensatory mechanisms. Concurrently, hepatic injury is known to induce hemoconcentration, thereby contributing to the elevated levels of TP and ALB [44]. Furthermore, liver damage activates the body’s immune response, leading to an augmented production of immunoglobulins, which in turn results in an increased GLB value.

**Fig. 9:**
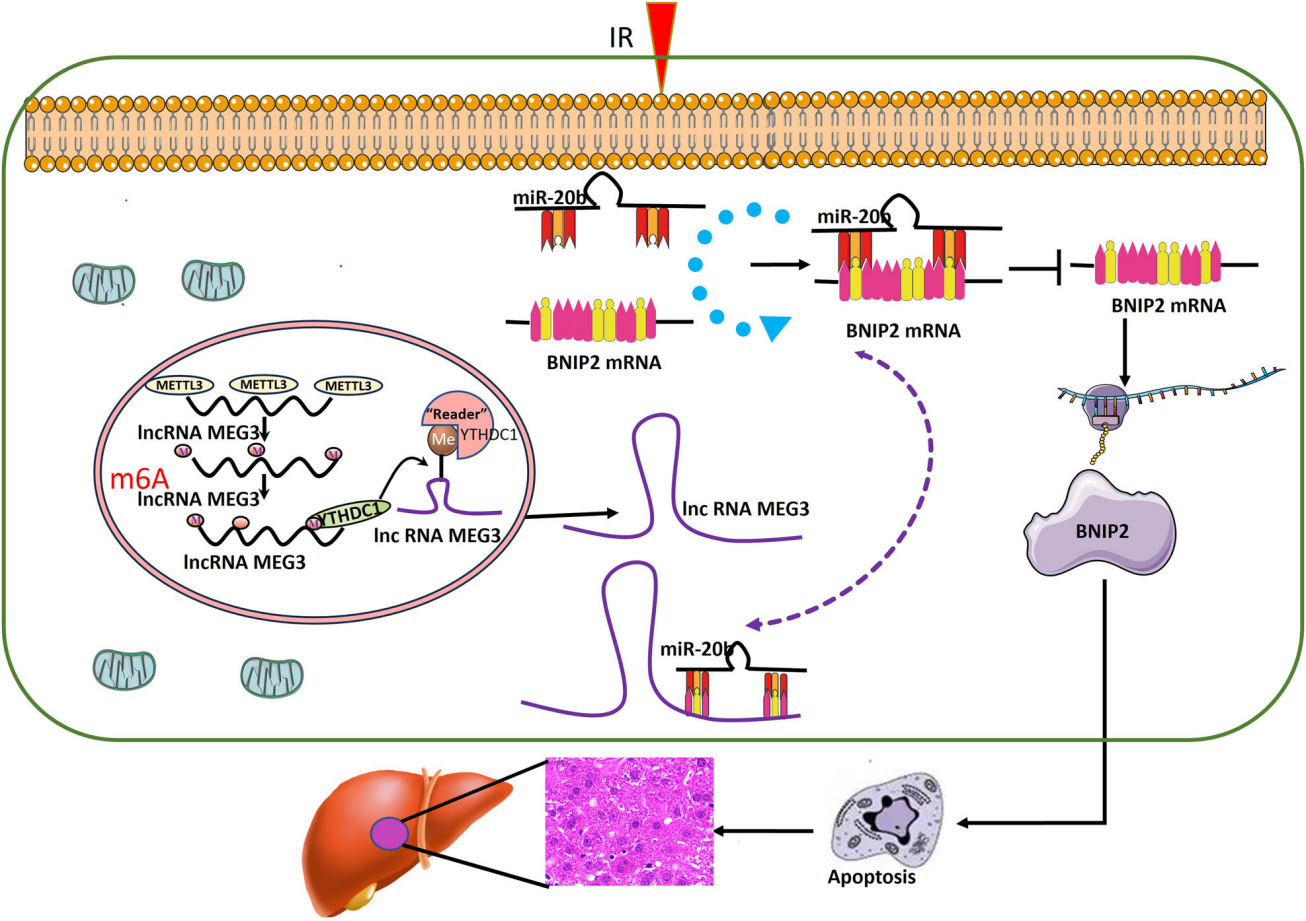
YTHDC1-dependent METTL3-mediated m^6^A modification of MEG3 early during RILI and consequent regulation of the miR-20b-mediated inhibition of BNIP2.

In conclusion, these results offer new insights into METTL3-mediated YTHDC1-dependent MEG3 m^6^A modification early during the progression of RILI, ultimately leading to regulations in the miR-20b-mediated inhibition of BNIP2 (Fig. 9). Additional detailed research efforts will be vital to fully elucidate the mechanisms that govern m^6^A modification patterns in RILI. First, to exert their biological effects, m^6^A modifications must be selectively recognized by specific interacting proteins [45], and multiple m^6^A readers may shape these biological processes [46]. Here, only YTHDC1 was analyzed, underscoring the need for studies of future regulatory functions for other reader proteins. Second, the importance of METTL14, WTAP, FTO, and ALKBH5 as regulators of m^6^A also warrants further study. Third, other candidates beyond MEG3 may play roles in the METTL3-mediated YHTDC1-dependent regulatory pathway elucidated herein, and more studies will be necessary to test this possibility.

## Supplementary information

supplementary figures

raw data

## Data Availability

The original data of this study are detailed in the Supplementary Material, and any further inquiries may be addressed to the corresponding author.
