# Supplementary material for: The YTHDC1 reader protein recognizes and regulates the lncRNA MEG3 following its METTL3-mediated m6A methylation: a novel mechanism early during radiation-induced liver injury
Source: Cell Death Dis. 2025 Feb 24;16(1):127. doi: 10.1038/s41419-025-07417-2 (PMC11850776; doi:10.1038/s41419-025-07417-2)
Supplement: Supplementary file 1 — supplementary figures [file 41419_2025_7417_MOESM1_ESM.docx]

**
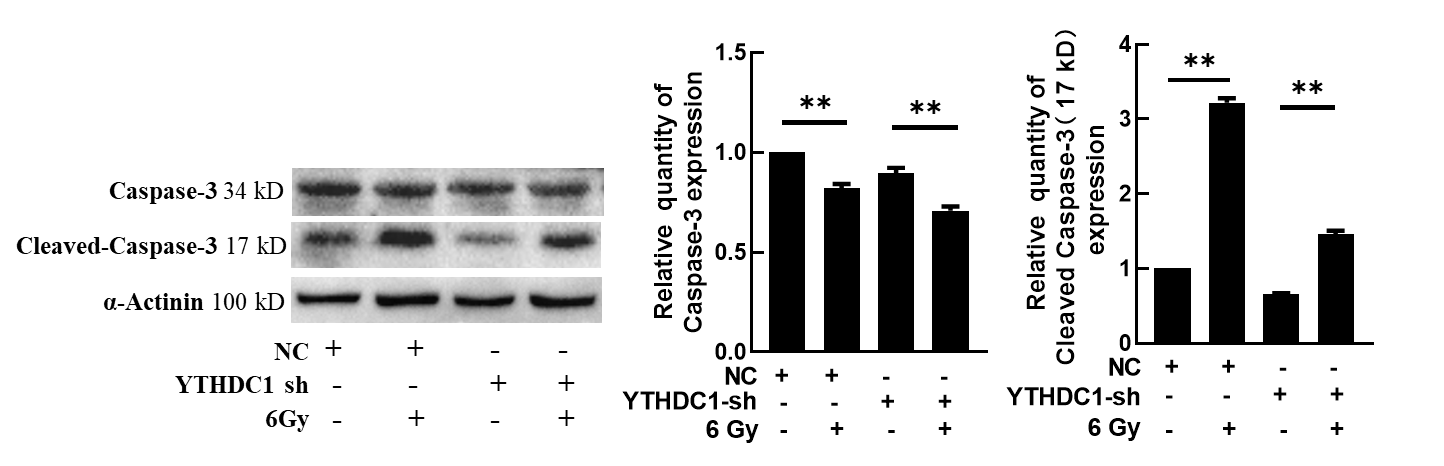
**

**S-Fig.1 The effects of silencing YTHDC1 on Caspase-3 protein in the liver of mice that were or were not irradiated (**, P<0.01).** n=8. It was performed by one-way ANOVA.

**
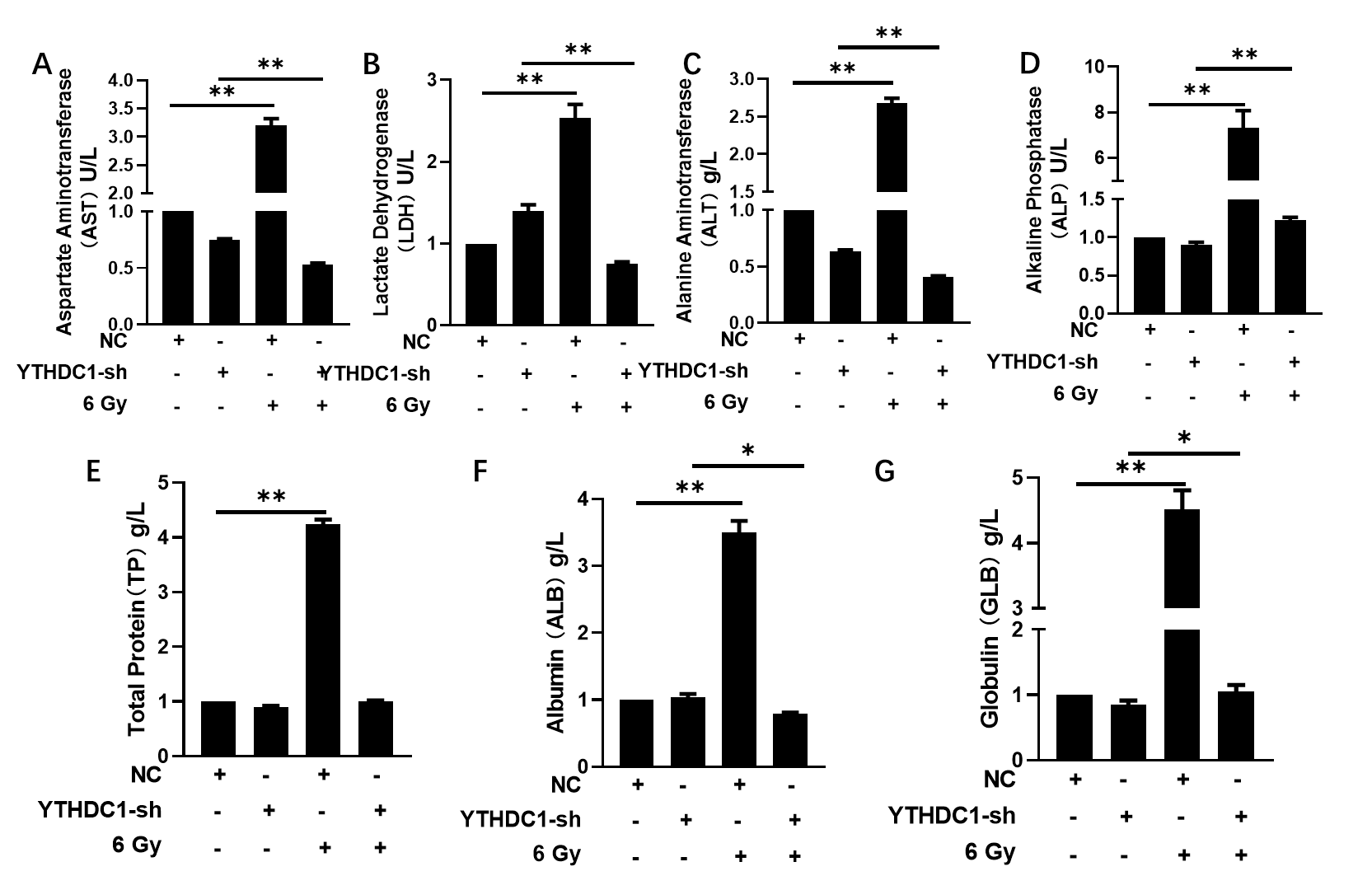
S-Fig.2 The impact of radiation on liver damage in YTHDC1 shRNA mice.** A. Expression levels of AST following radiation damage. B. LDH expression after exposure to radiation-induced. C. ALT expression in response to radiation damage. D. ALP expression resulting from radiation exposure. E. TP expression following radiation-induced injury. F. ALB expression after radiation damage. G. GLB expression in the context of radiation injury. **, P<0.01; *, P<0.05. A-G, n=8. A-G were performed by one-way ANOVA.

**
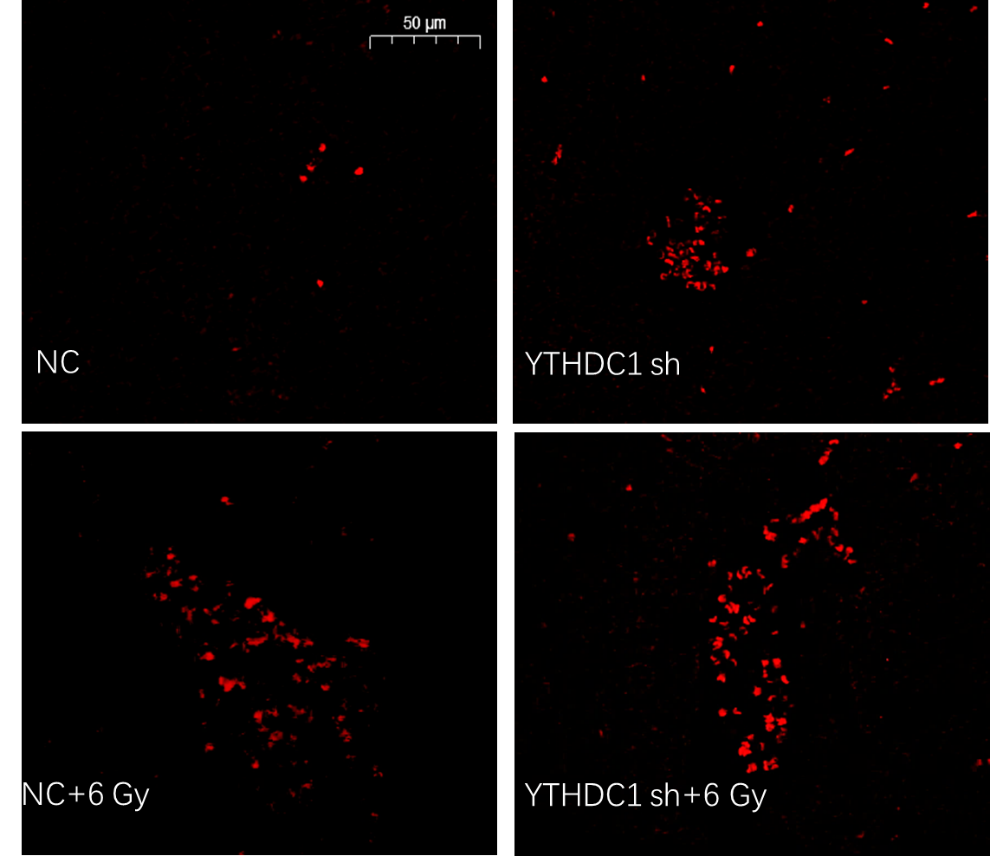
**

**S-Fig.3 Apoptosis in murine liver tissue was evaluated using TUNEL staining following the knockdown of YTHDC1 and irradiation induced damage.** Scale bar: 50 µm.
