# Supplementary figures and images for: The YTHDC1 reader protein recognizes and regulates the lncRNA MEG3 following its METTL3-mediated m6A methylation: a novel mechanism early during radiation-induced liver injury
Source: Cell Death Dis. 2025 Feb 24;16(1):127. doi: 10.1038/s41419-025-07417-2 (PMC11850776; doi:10.1038/s41419-025-07417-2)

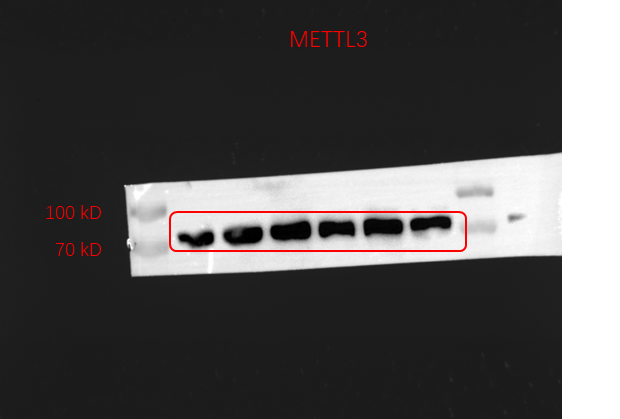


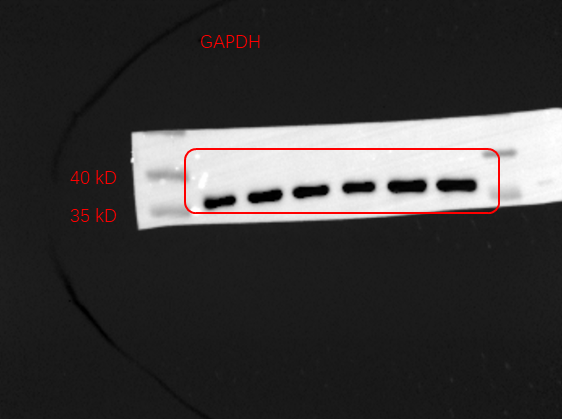


Fig.1-B


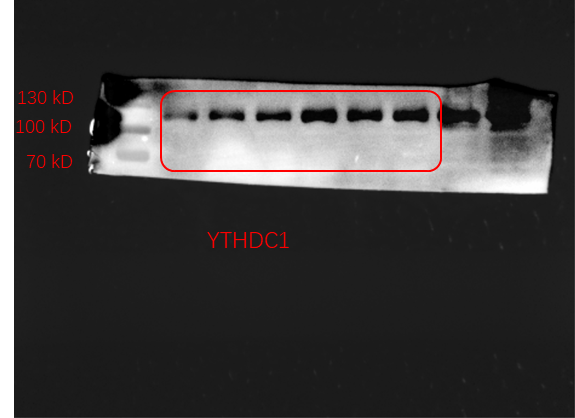


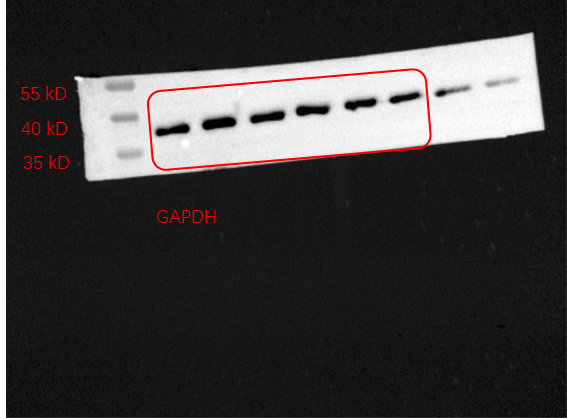


Fig.1-C


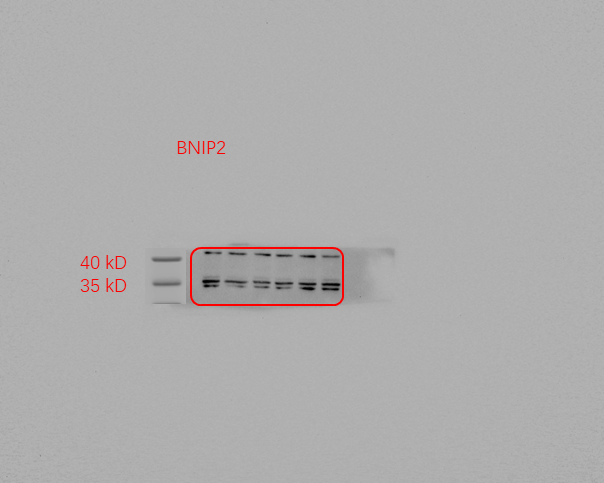


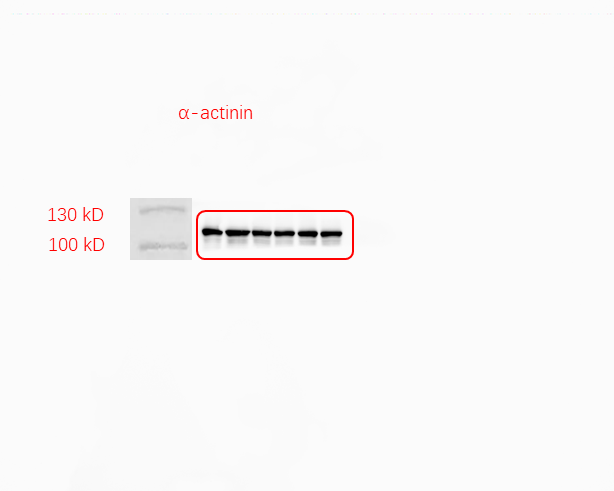


Fig.1-G


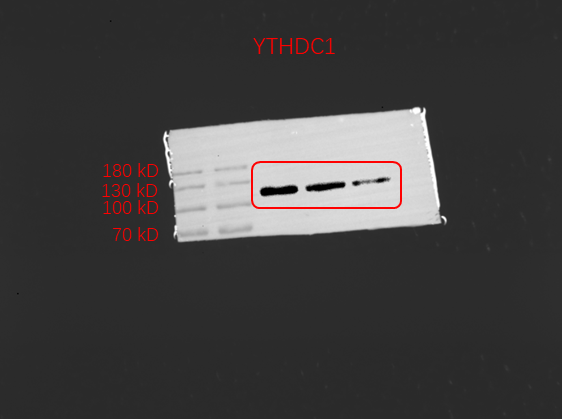


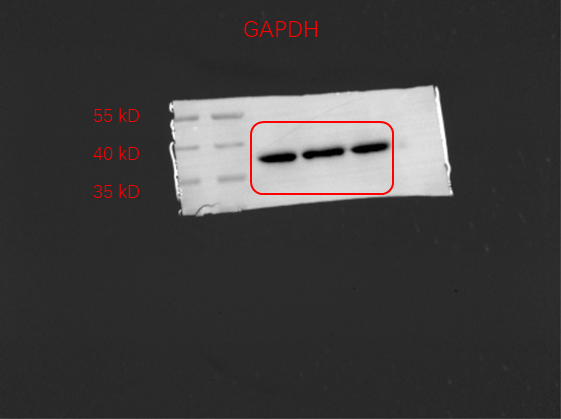


Fig.2-A


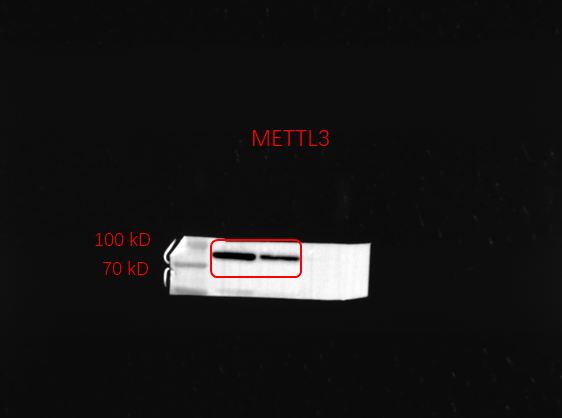


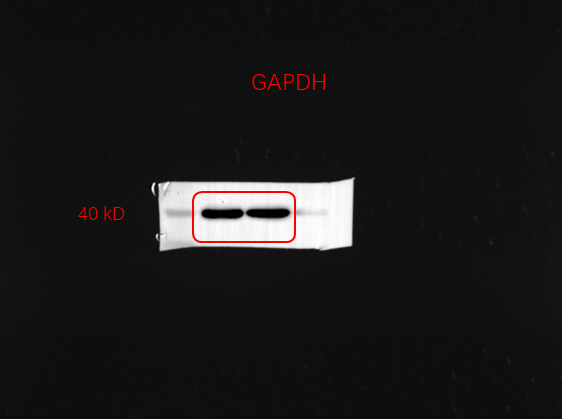


Fig.4-D


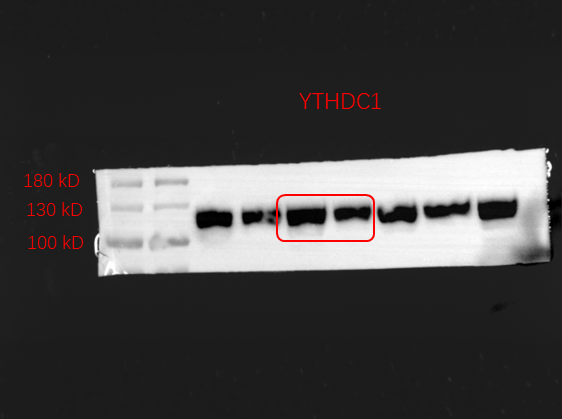


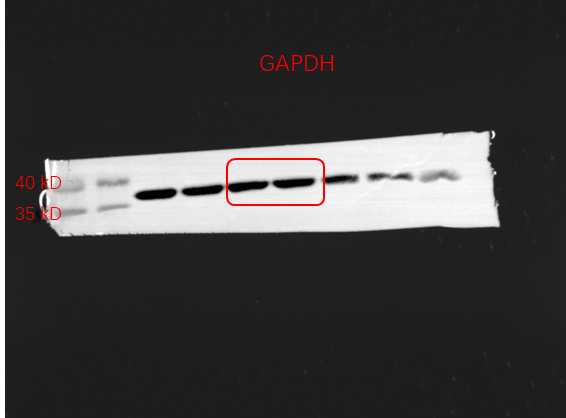


Fig.5-A


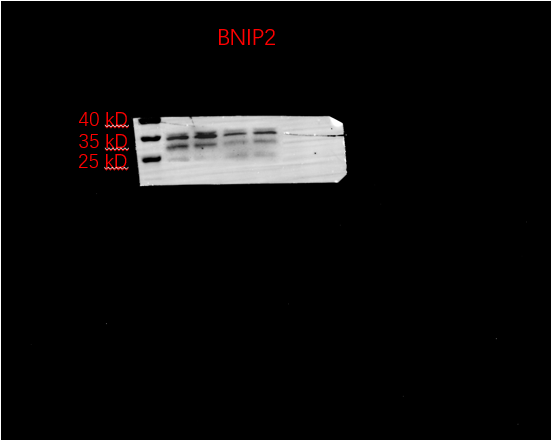


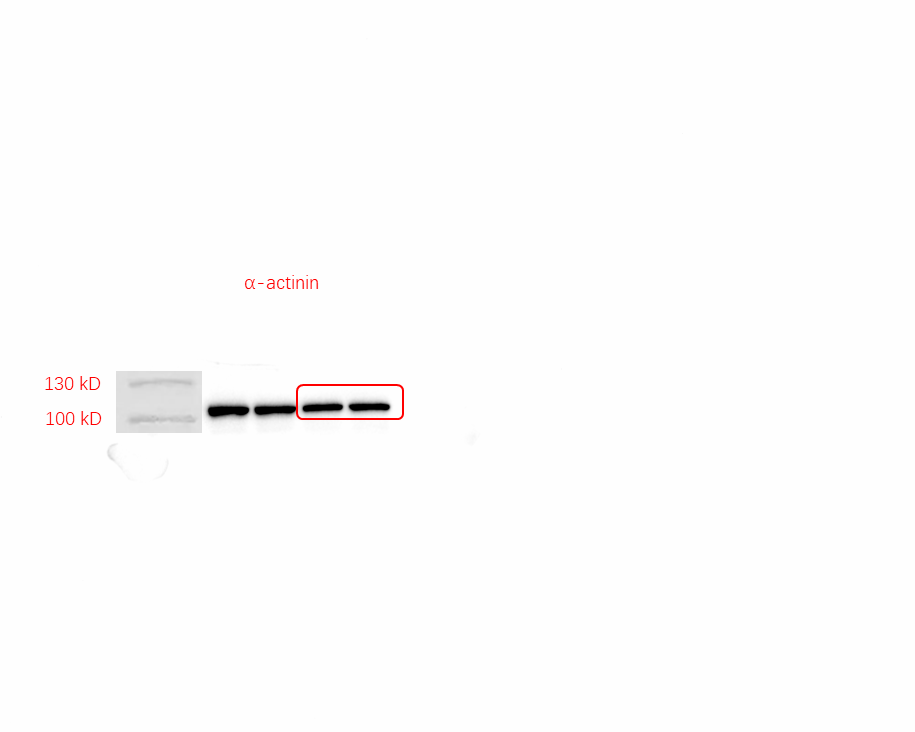


Fig.6-D


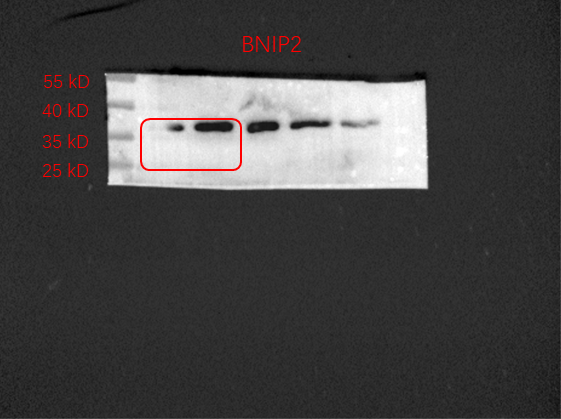


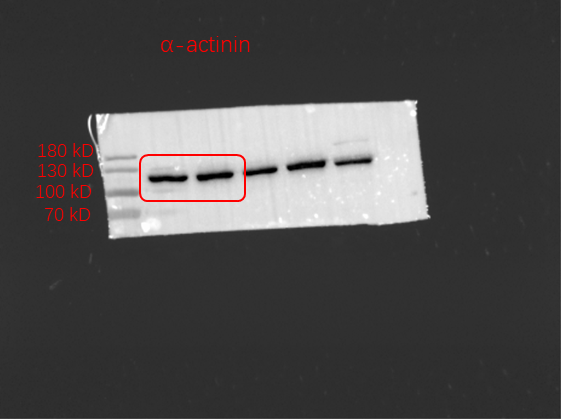


Fig.6-E


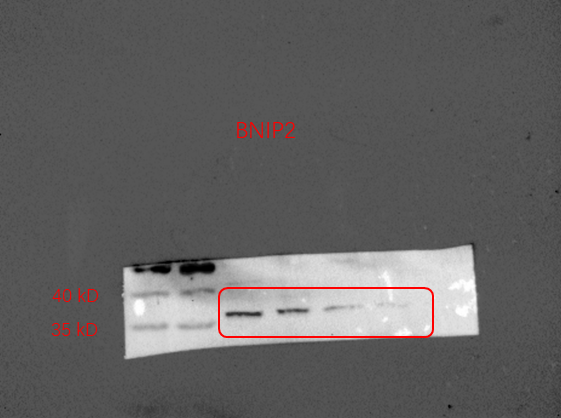


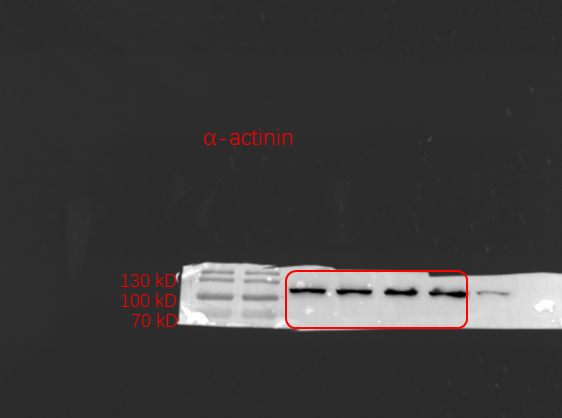


Fig.6-H


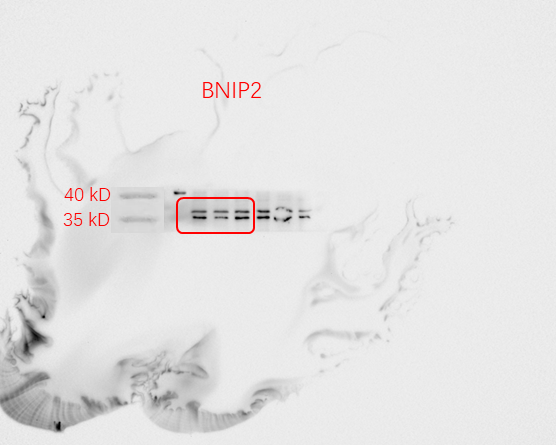


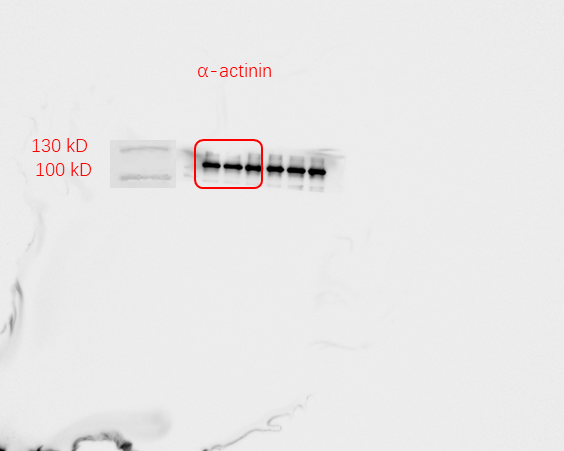


Fig.6-I


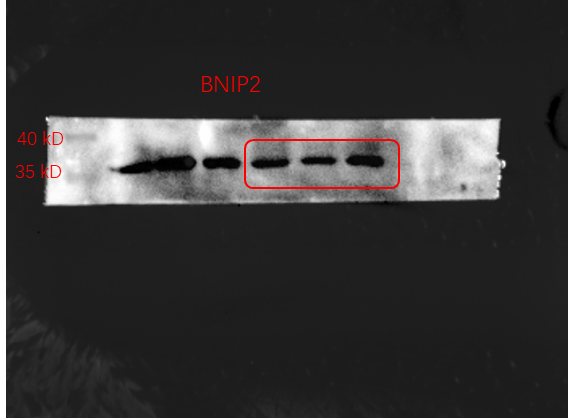


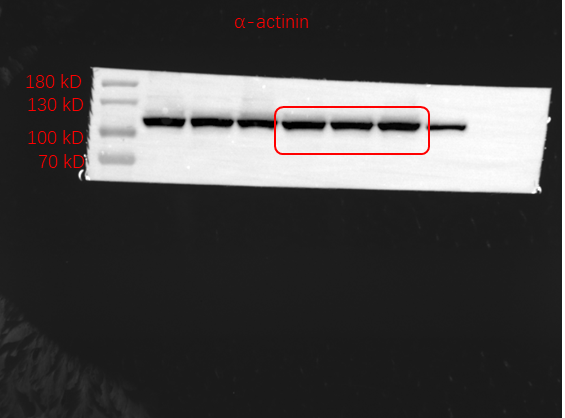


Fig.6-J


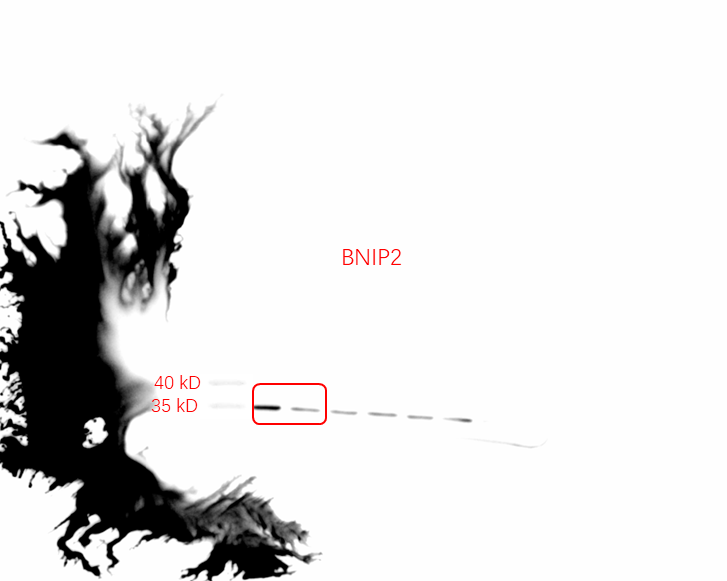


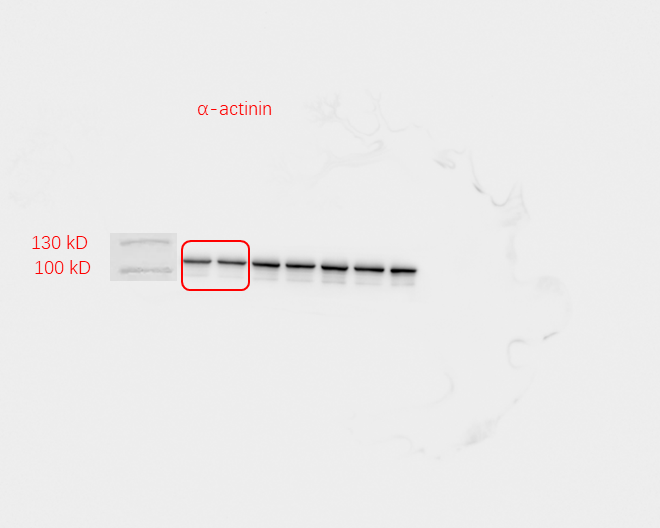


Fig.7-B


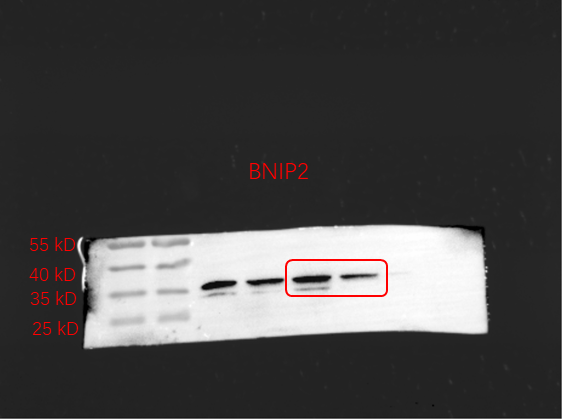


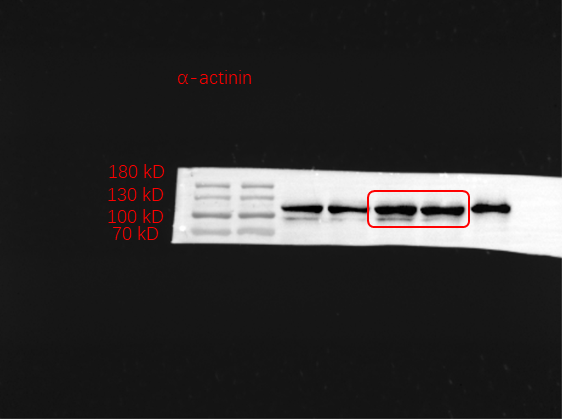


Fig.7-C


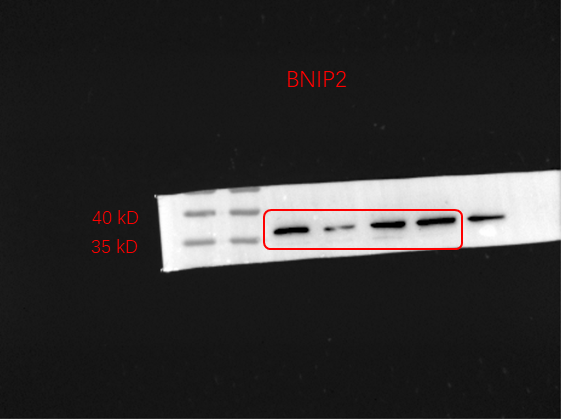


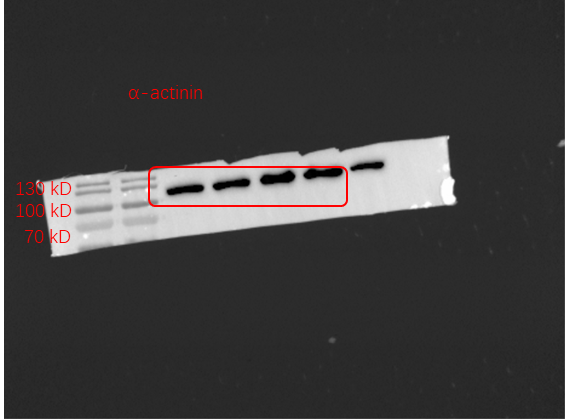


Fig.7-D


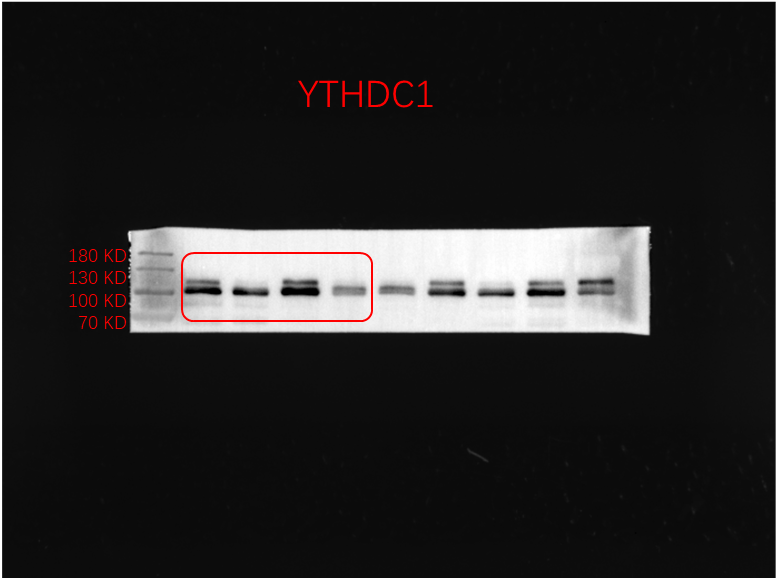


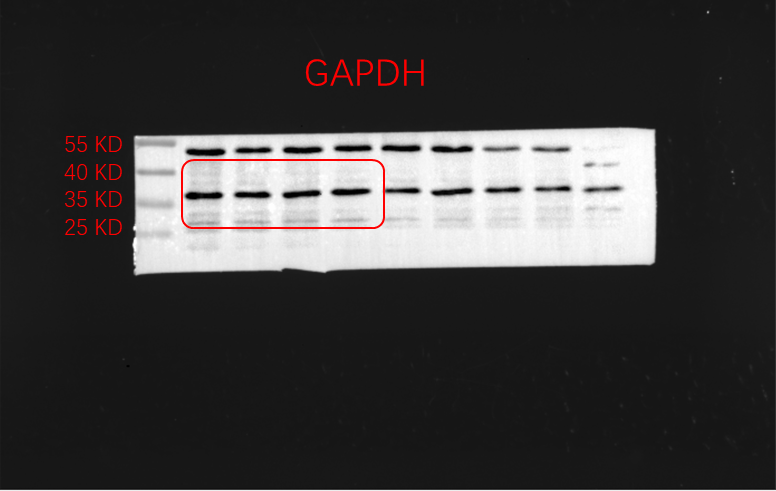


Fig.8-C


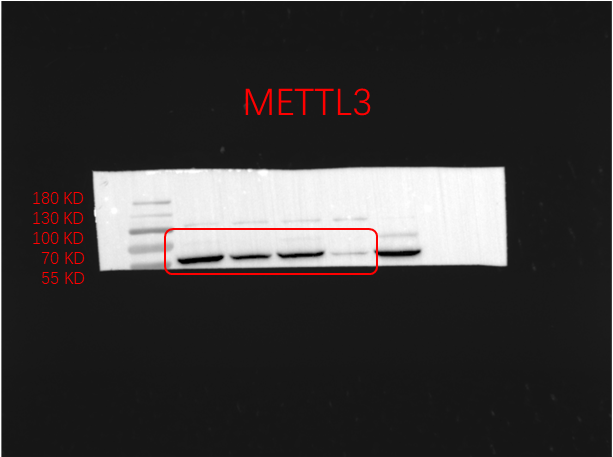


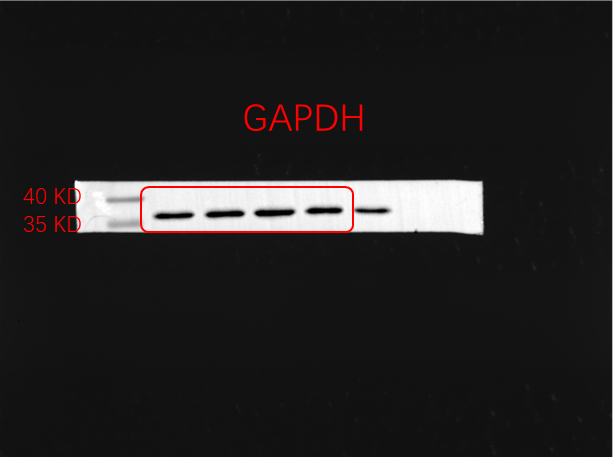


Fig.8-D


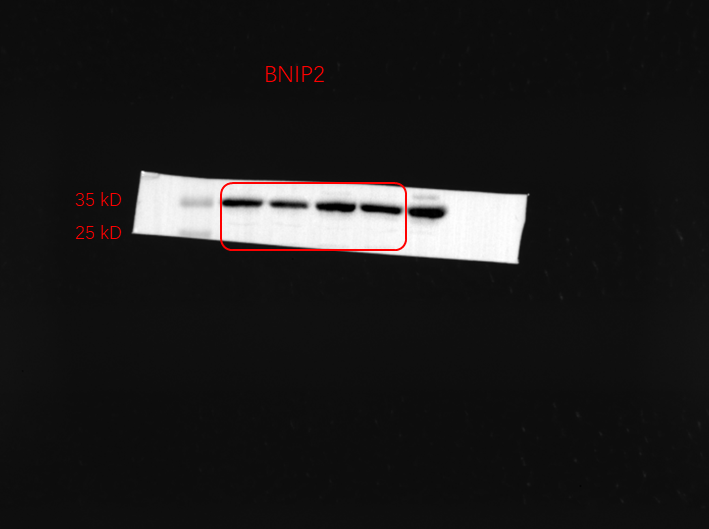


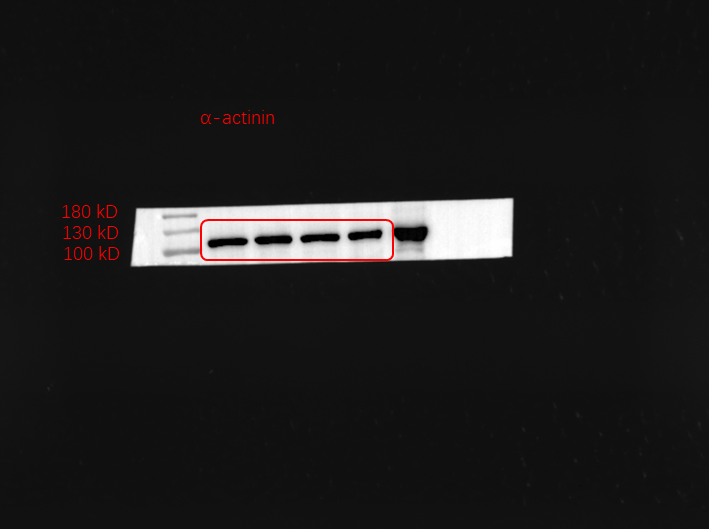


Fig.8-G


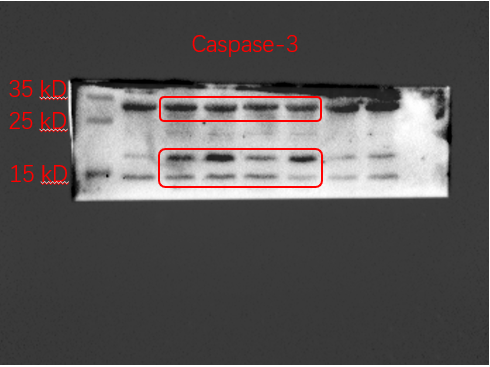

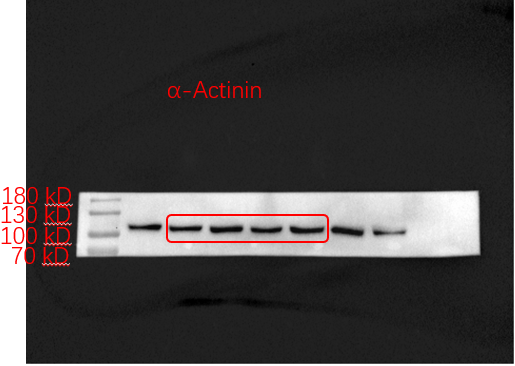


S-Fig.1

Supplement: Supplementary file 2 — raw data [file 41419_2025_7417_MOESM2_ESM.docx]
